# Low validity of Google Trends for behavioral forecasting of national suicide rates

**DOI:** 10.1371/journal.pone.0183149

**Published:** 2017-08-16

**Authors:** Ulrich S. Tran, Rita Andel, Thomas Niederkrotenthaler, Benedikt Till, Vladeta Ajdacic-Gross, Martin Voracek

**Affiliations:** 1 Department of Basic Psychological Research and Research Methods, School of Psychology, University of Vienna, Vienna, Austria; 2 Wiener Werkstaette for Suicide Research, Vienna, Austria; 3 Suicide Research Unit, Department of Social and Preventive Medicine, Center for Public Health, Medical University of Vienna, Vienna, Austria; 4 Psychiatric Hospital, University of Zurich, Zurich, Switzerland; University of Toronto, CANADA

## Abstract

Recent research suggests that search volumes of the most popular search engine worldwide, Google, provided via Google Trends, could be associated with national suicide rates in the USA, UK, and some Asian countries. However, search volumes have mostly been studied in an ad hoc fashion, without controls for spurious associations. This study evaluated the validity and utility of Google Trends search volumes for behavioral forecasting of suicide rates in the USA, Germany, Austria, and Switzerland. Suicide-related search terms were systematically collected and respective Google Trends search volumes evaluated for availability. Time spans covered 2004 to 2010 (USA, Switzerland) and 2004 to 2012 (Germany, Austria). Temporal associations of search volumes and suicide rates were investigated with time-series analyses that rigorously controlled for spurious associations. The number and reliability of analyzable search volume data increased with country size. Search volumes showed various temporal associations with suicide rates. However, associations differed both across and within countries and mostly followed no discernable patterns. The total number of significant associations roughly matched the number of expected Type I errors. These results suggest that the validity of Google Trends search volumes for behavioral forecasting of national suicide rates is low. The utility and validity of search volumes for the forecasting of suicide rates depend on two key assumptions (“the population that conducts searches consists mostly of individuals with suicidal ideation”, “suicide-related search behavior is strongly linked with suicidal behavior”). We discuss strands of evidence that these two assumptions are likely not met. Implications for future research with Google Trends in the context of suicide research are also discussed.

## Introduction

More than one third of the world population is using the Internet [1]. A high percentage of internet users in the USA use the Internet for news reports and to gather information about health. In the USA, 24% of users seek at least once per week information about health-related topics [2].

The prevalence rate of health-specific online searches is estimated at 4.5% of all online searches [3], but the true prevalence might be even higher. Google reported 1200 billion search requests worldwide in 2012 [4]. With a prevalence of 4.5% and 3.3 billion daily requests (in 2012), it can be estimated that approximately 148 million health-related search requests are made every day.

In 2012, 72% of US Internet users searched the Internet for health topics [5]. A majority of 77% utilized for their searches search engines like Google, Bing or Yahoo. The most popular search engine worldwide is Google. Its global usage share was up to 90% in the time span from 2010 to 2016 [6].

Death by suicide is in the top 10 causes of death in the USA [7] and in Europe (e.g., [8]). Suicide as a cause of death is epidemiologically important; hence, suicide prevention is an important goal of public health interventions and research. Previous studies have investigated the influence of the Internet on suicidal behavior (e.g., [9–11]) or specifically have focused on the use of search engines to gather information on suicide-related contents in the Internet [12–25]. Currently, the state of research (reviewed below) suggests that there might be associations between online searches and suicide rates. Thus, online search data might be useful in the surveillance and monitoring of suicidal behavior, and might inform national suicide-prevention efforts, e.g., [12]. Therefore, the objective of this study was to investigate whether Google Trends can be used as a reliable tool on an aggregate level for behavioral forecasting of national suicide rates.

### Google Trends

Google Trends is an analysis tool which was released in its original version by Google in May 2006 [26]. It provides information on the weekly request frequencies of search terms that can be specified by country, time span, category, and media for the period of January 2004 to the present week. Google Trends provides the total volume for requested search terms, normalized in a way that countries of different size can be compared [27]. Normalizing takes into account the total number of search requests for each country which itself is not reported. Reported normalized scores are values between 0 and 100. For insufficient data, the value 0 is displayed. The information is presented graphically, but also can be downloaded in CSV data format. For that purpose, registration and login is necessary. It is important to mention that the data provided by Google Trends allow no conclusions with regards to individual search behavior [27].

The exact algorithm used by Google Trends remains, for the most part, unknown. However, Google provides some explanations to their results. Search terms that are requested rarely and repeated requests of the same term from the same user within a specific (but not defined) time span are excluded from the analysis. The spelling of search terms is important as well. Terms that are searched with or without quotations marks can vary with regards to their outcome [27].

### Current state of research and present study goals

In the past few years, a number of studies have examined the utility of search engine query data for monitoring influenza activity and influenza epidemics [28–33]. One seminal contribution to this field of research was the study of Ginsberg and colleagues [30] who used Google search data for their analyses on the activity of influenza in the USA. Based on this study, Google Flu Trends was published, providing estimates on the activity of influenza all over the world based on query data [34]. Google Flu Trends received broad attention at that time, but was repeatedly criticized for lack of validity and reliability in following years [35–38]. Big data (for a recent account of definitions, applications, and methods of analysis in the field of psychology, see [39]) might not be reliable and valid for scientific use in general, because tools that provide big data (like Google Trends) were not created for scientific purposes; more specifically, the use of big data might also lead to problems of overfitting, when a large number of potential predictors (millions of search terms) are used to predict only a small number of cases [36]. In response to the critique of the scientific community, Google Flu Trends was revised repeatedly in 2009 and in 2013 [36,38]. It was finally discontinued and public access was restricted to historical data up to 2015, but Google still provides scientific partners with current data [34]. Google Trends is still publicly available and provides current data to anyone.

Search engine query data have since been used for various health-related topics, such as electronic nicotine delivery systems, noninfectious medical conditions, kidney stones or whiplash [40–45], and also in other fields of research, such as economics (e.g., [26,46]). The results of these studies indicated sometimes sizeable associations between query data and aggregate-level outcomes (ranging from *r* = .78 to *r* = .91 in [28–30,42,45]). However, some authors also cautioned on the validity of reported results and emphasized that independent verification and follow-up was needed (e.g., [43]).

Studies using search engine query data are fewer in the field of suicide research than in influenza research. However, the relevance of suicide-related topics for online searches is shown by the fact that, for example, the search term *depression* reached nearly 85% of the search frequency of the term *Barack Obama* during July 2008 to July 2009. In the same period, the term *suicide* reached nearly 80% of the frequency of the term *depression* [47].

The majority of previous studies on the associations of Google Trends search volumes with suicide rates originated from English-speaking countries, especially the USA [12–14] and the United Kingdom [15–17], but also from Asia [17–20]. Apart from suicide-related search terms (e.g., *suicide*, *commit suicide*), studies also have investigated search terms concerning specific methods (e.g., *suicide by jumping*, *hydrogen sulfide*) and selected risk factors of suicide (e.g., *depression*, *unemployment*). Characteristics and findings of previous studies are summarized in Table 1. The studies of Page et al. [21], Song et al. [22], and Arendt and Scherr [23] are omitted from this overview. Page et al. [21] presented data on associations of Google Trends search volumes with unemployment rates, but not with suicide rates. Song et al. [22] explored effects of yearly suicide rates on monthly Google Trends search volumes, but not of search volumes on suicide rates. Arendt and Scherr [23] provided evidence of seasonal, daily, and holiday-related peaks of *poisoning* search query volumes that appear to correspond to known fluctuations in suicide rates. However, they presented no analyses on a direct relationship of search volumes and suicide rates.

**Table 1:** Results of previous studies on the associations between Google Trends search volumes and suicide statistics.

Study	Country	Search terms	Method	Relevant results	Effect size
Ma-Kellams et al. (2016) [12]	USA	*suicide*, *how to suicide*, *how to kill yourself*, *how to commit suicide*, *painless suicide*	Correlation and linear regression analysis	Positive associations of national suicide rates with all terms except *suicide*	Large (*r* = .49 to .63)
Gunn & Lester (2013) [13]	USA	*commit suicide*, *suicide prevention*, *how to suicide*	Correlation analysis	Positive associations of national suicide rates with *commit suicide* and *suicide prevention*	Medium to large (*r* = .31 and .61)
McCarthy (2010) [14]	USA	*suicide*, *teen suicide*, *depression*, *divorce*, *unemployment*	Correlation analysis	Positive associations of national suicide rates and rates of intentional self-injury of 15–25 year-olds with *suicide*	Large (*r* = .70 and .50)
Kristoufek et al. (2016) [15]	UK	*suicide*, *depression*	Time-series analysis	Positive associations of national suicide rates with *suicide* at lags 0 and -1 and with *depression* at lags -5 to -10, negative associations with *suicide* at lags -6 to -11	Search volumes explained 22% of suicide rates
Bruckner et al. (2014) [16]	UK	*suicide*, *suicide and methods*, *suicide and depression*, *depression*, *depression and help*, *suicide and help*	Time-series analysis	Positive associations of suicide rates in England and Wales with *suicide and depression*, *depression*, and *depression and help* at lag 0	Search volumes explained 7% of suicide rates
Chang et al. (2011) [17]	UK & Japan	*suicide*, *hydrogen sulfide*	Graphical display	Increase of search requests after media reporting	N/A
Hagihara et al. (2012) [18]	Japan	*a suicide*, *sites on suicide*, *suicide methods*, *hydrogen sulfide*, *hydrogen sulfide suicide*, *suicide hydrogen sulfide*, *bulletin board system (BBS) on suicide*, *suicide rates*, *suicide by jumping*, *depression suicide*	Time-series analysis with transfer model and prewhitening	Positive associations of national suicide rates of 20–29 year-olds and 30–39 year-olds with *hydrogen sulfide*, *hydrogen sulfide suicide*, and *suicide hydrogen sulfide* at lag -11; as well as of the 30–39 year-olds with *suicide by jumping* at lag -6 und *BBS on suicide* at lag -5	N/A (unstandardized coefficients)
Sueki (2011) [19]	Japan	*suicide*, *depression*, *suicide method*	Cross-correlation analysis	Positive associations of national suicide rates with *depression* at lags -3 to 0, negative association at lag +3	Medium to large (*r* = .25 to .43)
Yang et al. (2011) [20]	Taiwan	37 search terms, including *major depression* and *suicide*	Cross-correlation and linear regression analysis	Positive associations of suicide rates in Taipei City with 18 search terms at lags -2 to 0	Medium to large (*r* = .27 to .48)

Notably, previous studies suffered from a number of important methodological shortcomings: first, studies selected search terms mostly in an ad hoc fashion and did not systematically evaluate for which terms Google Trends actually provides analyzable data. Yang et al. [20] investigated a larger pool of search terms, but only with the intent to exclude from further analysis those search terms that showed no significant cross-correlations with suicide rates.

Second, assessing the temporal associations between two time series may result in spurious findings when the analysis does not adequately account for structural similarities (e.g., autocorrelational pattern, temporal trends) in the explanatory time series (i.e., Google Trends search volumes) and the dependent time series (i.e., suicide rates). Similar autocorrelational patterns and trends may give rise to spurious associations for wholly independent time series. One method to adequately assess the associations (i.e., cross-correlations) of explanatory and dependent time series at various time lags is to model the explanatory time series with Box-Jenkins methods [48] and to utilize the fitted model as a transfer function model for the dependent time series. Associations are then examined for the residuals of the two time series, i.e., the prewhitened time-series data (see the Statistical Analysis section for details).

Most studies did not follow such an analysis plan. Chang et al. [17] provided only graphical displays. The US studies [12–14] reported Pearson correlations of explanatory and dependent time-series data. Sueki [19] and Yang et al. [20] assessed cross-correlations, but neither modeled the explanatory time series with Box-Jenkins methods, nor used transfer function models or performed prewhitening. Kristoufek et al. [15] and Bruckner et al. [16] used search volumes as predictors to model the dependent time series (i.e., suicide rates), but did not model the structural properties of the explanatory time series themselves. This may have spuriously inflated their importance in the time-series regression models. The study of Hagihara et al. [18] is an exception, as it used Box-Jenkins methods for the modeling of the explanatory time series, transfer function models and prewhitening. However, Hagihara et al. reported for their data associations of search volumes and suicide rates at lags 5 to 11 months prior to the suicide events; time spans that may be questionable from a psychological point of view with regards to the implied delays in suicidal behavior.

The current study had the following research goals: (1) to systematically collect relevant search terms in the field of suicide research, both with regards to pro-suicide terms and suicide prevention terms (see [13,16] in Table 1), and to evaluate respective Google Trends search volumes for availability and suitability for time-series analysis; (2) to conduct rigorous analyses on the temporal associations of search volumes and suicide rates, utilizing Box-Jenkins methods of time-series analysis, transfer function models and prewhitening; (3) to compare results for different countries, namely the USA, Germany, Austria, and Switzerland, in order to gain an impression of the generality of findings and their reproducibility across different countries, and to obtain first results for German-speaking countries for which evidence is currently lacking. Even though previous research investigated data from various countries (Table 1), this is to date the first and only study which directly compared results for different countries. With regards to Research Goal 2, we also examined possible variations across age groups and sex. Ultimately, the present study aimed at evaluating whether Google Trends can be considered a valid tool for behavioral forecasting of suicidal behavior on an aggregate level.

## Methods

### National suicide statistics data

US-American suicide statistics of the years 2004 to 2010 were retrieved from the website of the Centers for Disease Control and Prevention [49]. These data comprise all cases (244282 total, 192866 men, 51416 women) which have been diagnosed with X60-X84 (ICD-10), intentional self-harm [50], and cases which have been diagnosed with Y87.0, sequelae of intentional self-harm [51], or U03, a new code for terrorism within the category of suicide [52]. Some of the data do not contain information on subject age (0.01% female suicides and 0.03% male suicides). These data were included in the analyses on associations of search volumes with total suicide rates, and in separate analyses on associations of search volumes with suicide rates of men and women, but not in analyses on associations of search volumes with suicide rates of older men and women (see below).

German and Austrian data were retrieved from the respective official sources (Germany: DESTATIS, Austria: Statistik Austria). Both data sets comprised cases (Germany: 89282 total, 66361 men, 22921 women; Austria: 11744 total, 8935 men, 2809 women) diagnosed with X60-X84 (ICD-10) in the years 2004 to 2012. Data from Switzerland were retrieved from the national mortality database of the Swiss Bundesamt für Statistik, comprising cases (7635 total, 5486 men, 2149 women) diagnosed with X60-X84 (ICD-10) in the years 2004 to 2010. Switzerland was the only country which uses a specific code for assisted suicide which was recorded additionally to the ICD-10 diagnoses [53]. The cases of assisted suicides were separated from the other suicide cases and excluded from this research. Data collection complied with the terms of service for all respective websites.

### Google search terms

Selection of suicide-related search terms was done in a stepwise fashion. Authors RA, TN, and BT compiled a preliminary list of search terms that included the terms of previous studies [13,14,18,19,21] and which also drew on a study by Till and Niederkrotenthaler [25]. Search terms were classified into two groups: pro-suicide terms (such as *suicide*) and suicide prevention terms (such as *suicidal help*). The term *depression*, respectively the German equivalent *Depressionen*, was added to this list. The suicide mortality of depressed individuals is increased by a factor of 21 [54]. Additionally, some studies from different countries have reported associations of this term with suicide statistics [14,15,19,20]. The list can be found in S1 Appendix.

Terms were then entered into the regular Google search mask through a Firefox-based Tor browser (Version 24.6.0). Tor is a free, open-source software which encrypts and hides the location and other information of the user which are stored in cookies [55]. As a result, these data cannot be used by homepages or other services, which grants the user some anonymity. In the Tor browser, the country ID of the user can be freely selected. This allows the user to pretend to be located in, for instance, the USA, independently of his or her real location. In the current study, IDs in the Tor browser were matched to the respective countries for search requests to get a realistic impression of the actual search terms available in these countries.

Google search requests were made in the corresponding national language (i.e., English or German). German was also used for Switzerland that has four national languages (German, French, Italian, and Romansh [Rhaeto-Romanic]). German is spoken by a majority of 65% of the Swiss population [56]. Search terms consisting of more than one word were searched both with and without quotation marks. Search terms consisting of more than one word were also entered in different spellings and in different word orders to cover a broad spectrum of possibilities. Additional terms suggested by Google’s autocomplete function in these searches were amended to the list of search terms.

In a last step, we excluded search terms that pertained to movie titles, band or song titles, terms that are used in a lyrical or humorous context, scientific terms, and opaque or rare phrases. The revised list of search terms can be found in S2 Appendix.

### Google Trends time series

Google Trends time series were requested for the terms of the list for each country, restricting all requests to the time span of January 2004 to December 2013. This time span was used, because available suicide statistics spanned only the years 2004 to 2012, and time series’ asynchronicity (see next section) was somewhat decreased by specifying an explicit time span for the requested time series. In these requests, Google Trends also suggested related search terms. Terms suggested by Google Trends were collected and subsequently also requested from Google Trends. This procedure was iterated until Google Trends stopped suggesting new terms. Terms suggested by Google Trends can be found in S3 Appendix. Data collection complied with the terms of service for this website.

Based on the graphical monthly display provided on the Google Trends website, time series were categorized into one of four categories: (1) complete time series; (2) incomplete time series; (3) time series with single peaks, but (mostly) no further data; and (4) search terms without any data. For time series of the first category, alternative wordings, and singular and plural wordings were also tried. Requests with and without quotation marks were also performed if search terms contained more than one word. The categorization and further inspection of the weekly time-series data concerning length and completeness (see the Data preparation subsection of the Statistical Analysis section) resulted in the exclusion of several search terms (see S4 Appendix). Finally retained search terms had time series of Categories 1 or 2 and were used for further statistical analysis in this study. Retained search terms, and lengths (in months) and missing values (in weeks) of their respective time series are listed in Table 2. Notably, time series of suicide prevention terms could only be retained for the USA (*suicide help*, *“suicide help”*, *suicide hotline*, *“suicide hotline”*, *suicide prevention*, *“suicide prevention”*, *“suicide survivors”*), but not for other countries in this study.

**Table 2:** Final search terms and lengths (in months) and missing values (in weeks) of the respective Google Trends time series.

Country and search term	Length of time series (number of months)	Missing values
USA: pro-suicide terms		
*suicide*	01.2004–12.2010 (84)	0%
*depression*	01.2004–12.2010 (84)	0%
*how to kill yourself*	01.2005–12.2010 (72)	0%
*“how to kill yourself”*	05.2005–12.2010 (68)	1.69%
*how to overdose*	12.2005–12.2010 (61)	5.64%
*“how to overdose”*	12.2007–12.2010 (37)	0%
*online suicide*	02.2007–12.2010 (47)	2.93%
*painless suicide*	02.2004–12.2010 (83)	1.38%
*“painless suicide”*	05.2007–12.2010 (44)	5.73%
*“suicide chat”*	12.2006–12.2010 (49)	0%
*suicide methods*	02.2004–12.2010 (83)	0%
*“suicide methods”*	09.2004–12.2010 (76)	5.74%
USA: suicide prevention terms		
*suicide help*	02.2004–12.2010 (83)	1.37%
*“suicide help”*	11.2005–12.2010 (62)	5.19%
*suicide hotline*	02.2005–12.2010 (71)	0%
*“suicide hotline”*	05.2005–12.2010 (68)	3.04%
*suicide prevention*	01.2004–12.2010 (84)	0%
*“suicide prevention”*	01.2004–12.2010 (84)	0%
*“suicide survivors”*	02.2005–12.2010 (62)	0%
Germany		
*Suizid*	01.2004–12.2012 (108)	0%
*Selbstmord*	01.2004–12.2012 (108)	0%
*Depressionen*	01.2004–12.2012 (108)	0%
*Freitod*	11.2009–12.2012 (38)	0%
*Selbstmord Forum*	01.2004–12.2012 (108)	0%
Austria		
*Suizid*	12.2009–12.2012 (37)	4.94%
*Selbstmord*	11.2004–12.2012 (98)	4.92%
*Depressionen*	10.2006–12.2012 (75)	2.14%
Switzerland		
*Selbstmord*	01.2007–12.2010 (48)	2.39%
*Depressionen*	09.2007–12.2010 (40)	8.62%

#### Reliability of Google Trends time-series data

During the requests of Google Trends time-series data it became apparent that the request of time series for the same search term with the same self-selected regional and temporal specifications, but done on different days, resulted in (slightly) different and asynchronous results. Overall, time series were more asynchronous for earlier years than for later years. No information concerning this matter could be retrieved on the Google Trends website [27]. To minimize potential effects of asynchronicity of time series depending on the day of request, time-series data were requested for all final search terms for all countries on one and the same day (May 2, 2014).

In addition, for four search terms that were chosen on substantive grounds, daily requests were made from April 18, 2014 until April 27, 2014, thus yielding 10 time series per search term. These search terms included *depression*, *suicide manual*, *suicide methods* and *suicide* in English, and *Depressionen*, *Selbstmord*, *Selbstmordmethoden* and *Suizid* in German. Table 3 lists the reliabilities (intraclass correlation coefficients; two-way random model, assessing consistency of individual values) for averaged and individual time series of search terms *suicide* and *depression* (*Suizid* or *Selbstmord* and *Depressionen* in German) for which results of all four countries could be retrieved (see Table 2) and directly compared. As expected, averaged time series had higher reliabilities than individual time series (Table 3). Reliability of individual time series was also higher for larger countries (confidence intervals of coefficients overlapped for Austria and Switzerland). These averaged time-series data were used in supplemental analyses pertaining to these search terms and compared with the results of individual time-series data.

**Table 3:** Reliability of individual and averaged time series for selected Google Trends search terms.

Country and search term	Individual time series	Averaged time series
USA		
*suicide*	.991 [.990–.992]	.999 [.999–.999]
*depression*	.994 [.994–.995]	.999 [.999–1.000]
Germany		
*Suizid*	.91 [.90–.92]	.990 [.989–.991]
*Selbstmord*	.95 [.95–.96]	.995 [.994–.996]
*Depressionen*	.92 [.91–.93]	.991 [.990–.992]
Austria		
*Suizid*	.77 [.75–.80]	.97 [.97–.98]
*Selbstmord*	.74 [.72–.77]	.97 [.96–.97]
*Depressionen*	.63 [.60–.66]	.95 [.94–.95]
Switzerland		
*Selbstmord*	.78 [.76–.80]	.97 [.97–.98]
*Depressionen*	.68 [.65–.71]	.95 [.95–.96]

Numbers are intraclass correlation coefficients (two-way random model, assessing consistency of individual values) with 95% confidence intervals in brackets. All *p*s < .001.

### Statistical analysis

#### Analysis design

Younger individuals use the Internet more than older individuals, and older men use the Internet more than older women [2,57]. Higher age and male sex are known risk factors for suicide. Some previous studies also reported associations of search volumes with suicide rates of younger individuals [14,18]. Therefore, associations of search volumes with suicide statistics were investigated per country with (1) total suicide rates, suicide rates of (2) younger individuals and (3) older individuals, and with suicide rates of (4) older men and (5) older women.

Furthermore, poisoning is a frequent method of suicide among women [58]. Therefore, associations of US search volumes of *how to overdose* and *“how to overdose”* with overall suicide rates of men and women were also investigated.

In the data of the present study, the overall mean age of suicide was 40 years. This number was utilized for the separation of younger (<40 years of age) and older suicides (40+ years of age). Previous studies defined age brackets of 15–25 years [14] or 20–29 and 30–39 years [18]. For the USA, the previously reported median age of suicide was 46 years (based on 2003–2008 data from Virginia, USA [59]).

#### Data preparation

Google Trends weekly time-series data were downloaded in CSV data format. Time series were cropped at the start and at the end when they contained missing values at the margins. As suicide statistics were in a monthly format, and the unit of analysis was set to months, the weekly Google Trends time-series data were aggregated into monthly time series, using weighted averaging.

Finally retained time series (see Table 2) needed to span over at least 36 months (3 years), and were allowed a maximum of only 10% missing values in their original weekly resolution, of which at most 10 could be consecutive (i.e., spanned at most 2 months). Missing values in the weekly format of the time-series data were linearly interpolated before aggregation. It is recommended that for ARIMA modeling, time series should have 50 or more data points [60]. However, in order to be able to include data from Switzerland and some time series of the USA, Germany, and Austria in analysis, the limit was set to 36 data points (3 years). In total, 8 of 29 time series in the current study had less than 50 data points, the shortest time series had 37 data points (US search volumes on *“how to overdose”*; Table 2). The possible methodological shortcoming of investigating some time series with less than 50 data points was offset in the current study by focusing only on a narrow bracket of time lags in the cross-correlation analysis (see below).

#### Associations of search volumes with suicide rates

To investigate associations of search volumes with suicide rates, transfer function models of time-series analysis were used in this study. Transfer function models estimate to what extent changes in one time series are attributable to changes in one or more other time series [61]. In a first step, this required the modeling of the explanatory time series (i.e., search volumes), in order to perform in a second step the prewhitening on the explanatory time series and the dependent time series (i.e., suicide rates). For prewhitened explanatory and dependent time series the cross-correlation function was then determined in a third step. The three steps of analysis are described in more detail in the following.

Modeling of the explanatory time-series data required fitting of autoregressive integrated moving average (ARIMA) models, which are based on the methods of Box and Jenkins [47], to the individual search volume time series. For time series with seasonal components, seasonal ARIMA (SARIMA) models were used [62]. To find the adequate model for each time series, three steps were run iteratively [63]: the first step comprised the identification of the basic model, for which the autocorrelation functions (ACF) and the partial autocorrelation functions (PACF) of the data were inspected. The second step comprised model estimation, wherein all parameters of components of the model that had been identified in the previous step were tested for significance [64]. The last step comprised model diagnostics, wherein plots of the standardized residuals, the ACFs of the residuals, and Q-Q plots of the standardized residuals were graphically inspected. Well-fitting models (see [62]) had (1) significant (*p* < .05) model parameters; (2) residuals that scattered around zero and which on average did not differ significantly from null; (3) no remaining autocorrelation in the residuals (Ljung-Box-Pierce statistic was not significant).

For the detection of outliers, the method of Cryer and Chan [60] was used. Outliers were classified either as innovative (IO) or additive outliers (AO). Additive outliers affect only a single observation, while innovative outliers affect all later observations. If an outlier was detected, it was integrated by using an ARIMAX model (autoregressive integrated moving average model with explanatory variables).

If two or more alternative models appeared to fit the individual time-series data equally well, the model with lower AIC (Akaike Information Criterion), AICc (Akaike Information Criterion corrected), and BIC (Bayesian Information Criterion) values was chosen as the final model. Additionally, models with a simpler structure were preferred over models with a more complex structure [62]. For the preparation of data for time-series analysis and the fitting of ARIMA and ARIMAX models, the R packages TSA (Time Series Analysis) [65], zoo [66], gdata [67], and TSAgg [68] were used. For the fitting of SARIMA models, the R script itall.R [69] was used.

Prewhitening is used to guard against spurious correlations in transfer models of time-series analysis [63]. It is performed by finding the best-fitting model for the explanatory time series and using this specific model also for the dependent time series. The residuals of the explanatory time series may be considered white noise, but the residuals of dependent time series may have some pattern left [60]. If the residuals of the prewhitened and of the dependent time series are still correlated, one may conclude that changes in one time series can be attributed to changes in the other time series and were not merely caused by similar temporal processes underlying both time series in the same way [61]. The prewhiten function, which is included in the TSA package [64], was used for prewhitening. This function prewhitens the two time series, computes the cross-correlation function (see below), and plots the relevant data in one step.

The cross-correlation function (CCF) was used to assess the correlation between the prewhitened explanatory and dependent time series. To determine at which lags the explanatory time series worked as a predictor for the dependent time series, both synchronous (lag = 0) and asynchronous cross-correlations (lag ≠ 0) were computed. Asynchronous cross-correlations are the correlations between a time series *x* at time *t* and a second time series *y* at time *t*–*j*, where *j* denotes lag [63]. Significant cross-correlations with a negative lag imply that changes in the explanatory time series (*x*) lead to changes in the dependent time series (*y*) after *j* lags (“*x* leads *y*”). A positive lag implies that *y* influences *x* after *j* lags (“*x* lags *y*”) [62]. Lags -3 to +3 were considered relevant in this study (see next section).

#### Relevant time lags and their interpretation

Interpretation of significant cross-correlations at various time lags broadly followed a study by Sueki [19]: significant cross-correlations at negative lags indicate that the gathering of online information influenced later suicide statistics; i.e., by increasing (positive coefficient) or decreasing (negative coefficient) the probability of a suicide among individuals at risk who partook in the searches. For purposes of suicide behavior forecasting, cross-correlations at negative lags appear the most interesting; the current study therefore set a focus on these cross-correlations in the interpretation of results. If a simple causal relationship exists, pro-suicide search volumes should be positively associated with suicide rates at negative lags, whereas suicide prevention terms negatively.

Conversely, significant cross-correlations at positive lags indicate that suicide events increased or decreased later online searching behavior. Significant cross-correlations at lag zero are ambiguous with regards to these interpretations, as they lack any information on the exact temporal precedence of events; they merely allow the interpretation that events coincided.

With regards to the range of interpreted time lags in this study it appears increasingly hard to assume, and to prove empirically, that suicides and online searching behavior for suicide-related content may be associated across more than a few months (either preceding or following the suicide event). In line with a number of other studies using search query data [19,20,30,32,44,70–75], only the lags -3 to +3 (covering 3 months before, and 3 months after the suicide events) were thus considered relevant in the current study.

#### Expected cross-correlational patterns

On the premise that search volumes allow the forecasting of suicide rates, we expected that at negative lags pro-suicide terms and *depression* should be positively associated with suicide rates, whereas suicide prevention terms negatively. Building on effect sizes (Table 1) and findings of prior research (see Introduction and the Analysis design section), we hypothesized that overall cross-correlations should be of a large magnitude (*r* = .40), but larger for associations with suicide rates of younger individuals (*r* = .50) than older individuals (*r* = .40), and larger for associations with suicide rates of older men (*r* = .50) than older women (*r* = .40). In the case of search volumes for *how to overdose* and”*how to overdose”* we expected a larger magnitude for associations with suicide rates of women (*r* = .50) than men (*r* = .40). Finally, we assumed that cross-correlations should be largest at lag 0, decreasing in magnitude with each preceding lag in steps of .10.

## Results

### Fitted models

Table 4 presents the types of (S)ARIMA models that were fitted to the individual and averaged search volumes time series, and information on outliers in these data. Most of the models contained a yearly seasonal component, but otherwise had a relatively simple structure (low order of autoregressive, differencing, and moving average parts of the models; see table notes of Table 4). Fitted models for time series in an individual and averaged format were broadly similar.

**Table 4:** Fitted models and outliers in the individual and averaged Google Trends search volume time series per country.

Country	Search term	Fitted model	Type and position of outliers
USA	*suicide*	SARIMA(1,0,0)x(0,1,1)_12_	
		SARIMA(1,0,0)x(0,1,1)_12_	
	*depression*	SARIMA(0,1,2)x(1,0,1)_12_	IO: 56
		SARIMA(0,1,2)x(1,0,1)_12_	IO: 44, 56
	*how to kill yourself*	AR(1)	
	*"how to kill yourself"*	AR(1)	IO: 41
	*how to overdose*	SARIMA(0,1,1)x(0,1,1)_12_	
	*"how to overdose"*	SARIMA(1,1,0)x(1,0,0)_12_	
	*online suicide*	SARIMA(0,1,1)x(0,1,1)_12_	IO: 9, 21
	*painless suicide*	ARIMA(1,1,0)	IO: 52; AO: 5
	*"painless suicide"*	AR(1)	
	*"suicide chat"*	ARIMA(0,1,1)	
	*suicide methods*	ARIMA(1,1,0)	
		AR(1)	
	*"suicide methods"*	ARIMA(1,1,0)	
	*suicide help*	SARIMA(4,1,0)x(0,1,1)_12_	
	*"suicide help"*	SARIMA(0,1,1)x(1,0,0)_12_	
	*suicide hotline*	SARIMA(1,0,0)x(1,0,0)_12_	
	*"suicide hotline"*	AR(1)	
	*suicide prevention*	SARIMA(0,1,1)x(0,1,1)_12_	IO: 10
	*"suicide prevention"*	SARIMA(0,1,1)x(0,1,1)_12_	
	*"suicide survivors"*	SARIMA(0,1,1)x(0,1,1)_12_	
Germany	*Suizid*	ARIMA(0,1,1)	IO: 70, 93
		SARIMA(0,1,1)x(0,1,1)_12_	IO: 58
	*Selbstmord*	SARIMA(0,1,1)x(0,1,1)_12_	IO: 58, 70; AO: 95
		ARIMA(0,1,1)	IO: 70, 88, 103, 107; AO: 71
	*Depressionen*	SARIMA(0,1,1)x(0,1,1)_12_	IO: 58, 70; AO: 57
		SARIMA(0,1,1)x(0,1,1)_12_	IO: 58, 59, 70, 71, 72
	*Freitod*	ARIMA(0,1,1)	AO: 1
	*Selbstmord Forum*	ARIMA(0,1,2)	IO: 11; AO: 6
Austria	*Suizid*	AR(1)	
	*Selbstmord*	ARIMA(0,1,1)	IO: 86; AO: 87
		AR(1)	IO: 2, 3, 7, 9, 12; AO: 85
	*Depressionen*	ARIMA(0,1,1)	IO: 9
		MA(1)	IO: 8; AO: 11
Switzerland	*Selbstmord*	ARIMA(0,1,1)	
		AR(1)	
	*Depressionen*	ARIMA(1,1,0)	
		AR(1)	

Fitted models and outliers of averaged time series underlined. AR = autoregressive model, MA = moving average model, ARIMA = integrated autoregressive moving average model, SARIMA = seasonal ARIMA model. Parameters of the AR(*p*), MA(*q*), ARIMA(*p*,*d*,*q*), and SARIMA(*p*,*d*,*q*)x(*P*,*D*,*Q*)_*s*_ models denote: *p* = order of the autoregressive model (number of past time lags that affect current values autoregressively), *d* = degree of differencing (number of times the data had past values subtracted to reduce non-stationarity in the time series), *q* = order of the moving average model (number of current and past white noise error terms that affect current values), *P*,*D*,*Q* = autoregressive, differencing, and moving average terms of the seasonal part of the ARIMA model, *s* = number of periods in each season. Outliers were detected and classified as IO (innovative outlier) and AO (additive outlier) using the method of Cryer and Chan [60] and integrated into the models using ARIMAX modeling.

### Cross-correlations

#### Individual time-series data

**Fig 1:**
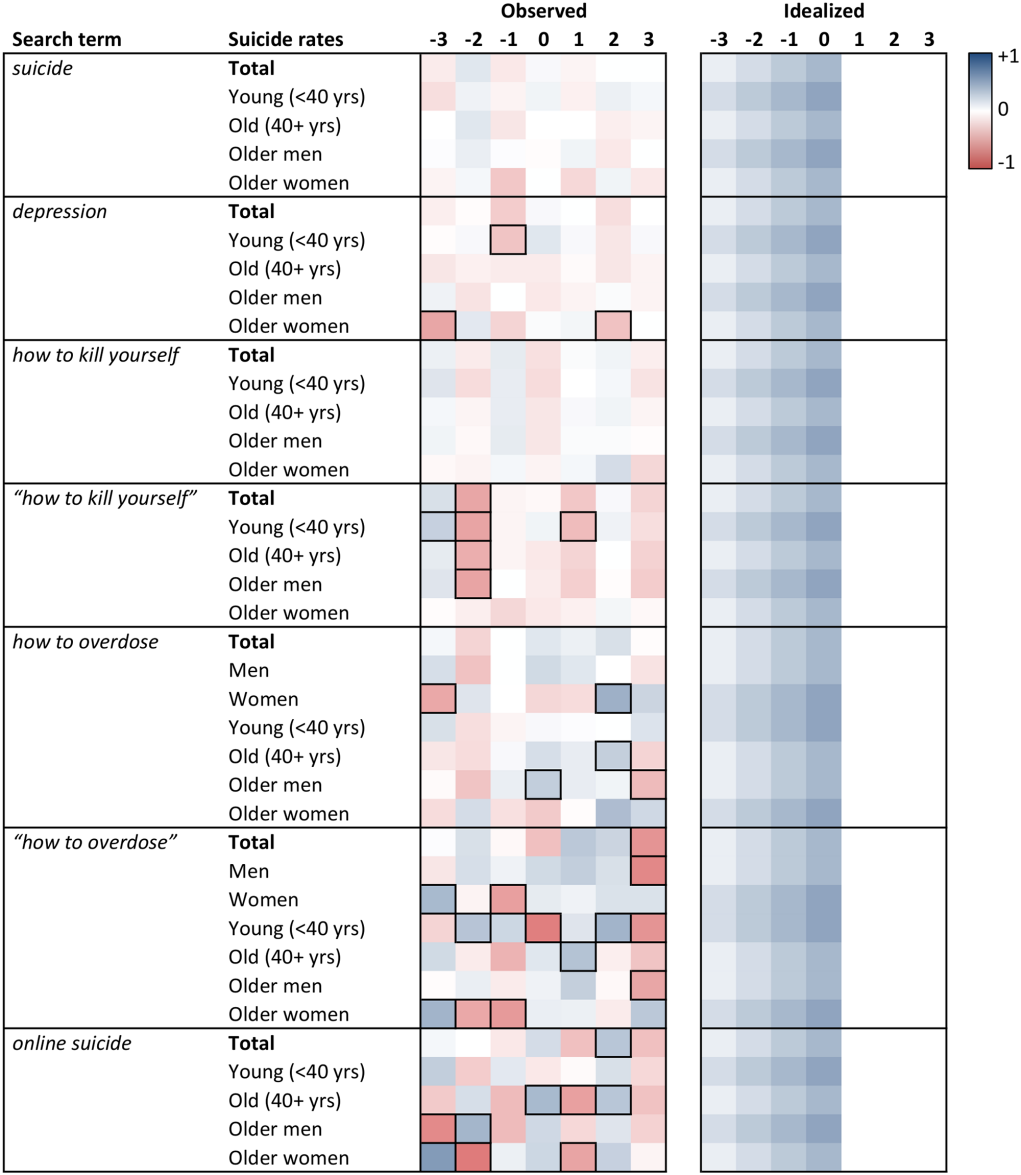
Heat map of cross-correlation coefficients for the US data (pro-suicide terms A). Numbers in column headers refer to lags in months. Idealized patterns reflect what could be expected if search volumes predicted suicide rates. In the observed patterns, frames highlight statistically significant (*p* < .05) coefficients.

**Fig 2:**
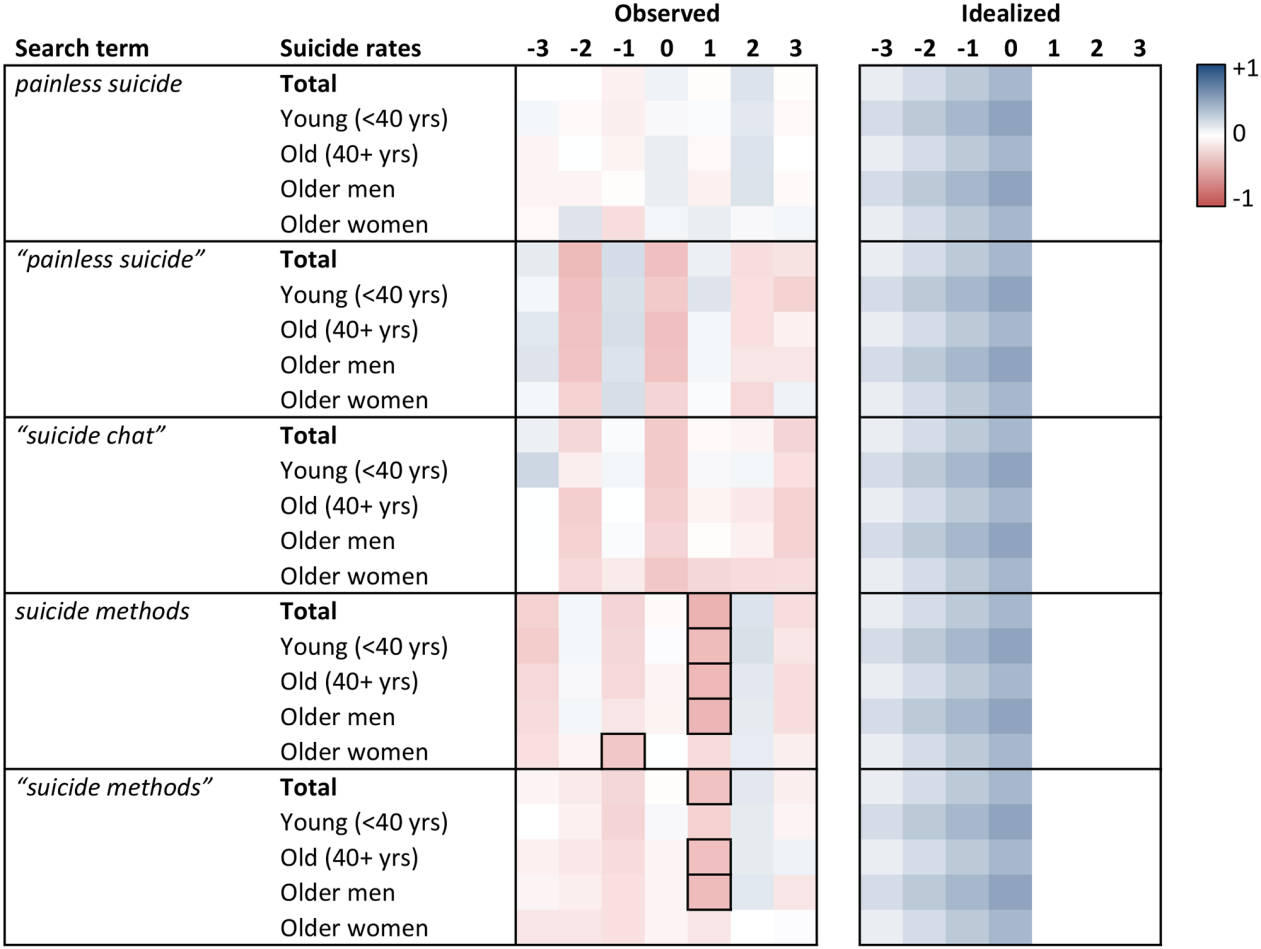
Heat map of cross-correlation coefficients for the US data (pro-suicide terms B). Numbers in column headers refer to lags in months. Idealized patterns reflect what could be expected if search volumes predicted suicide rates. In the observed patterns, frames highlight statistically significant (*p* < .05) coefficients.

**Fig 3:**
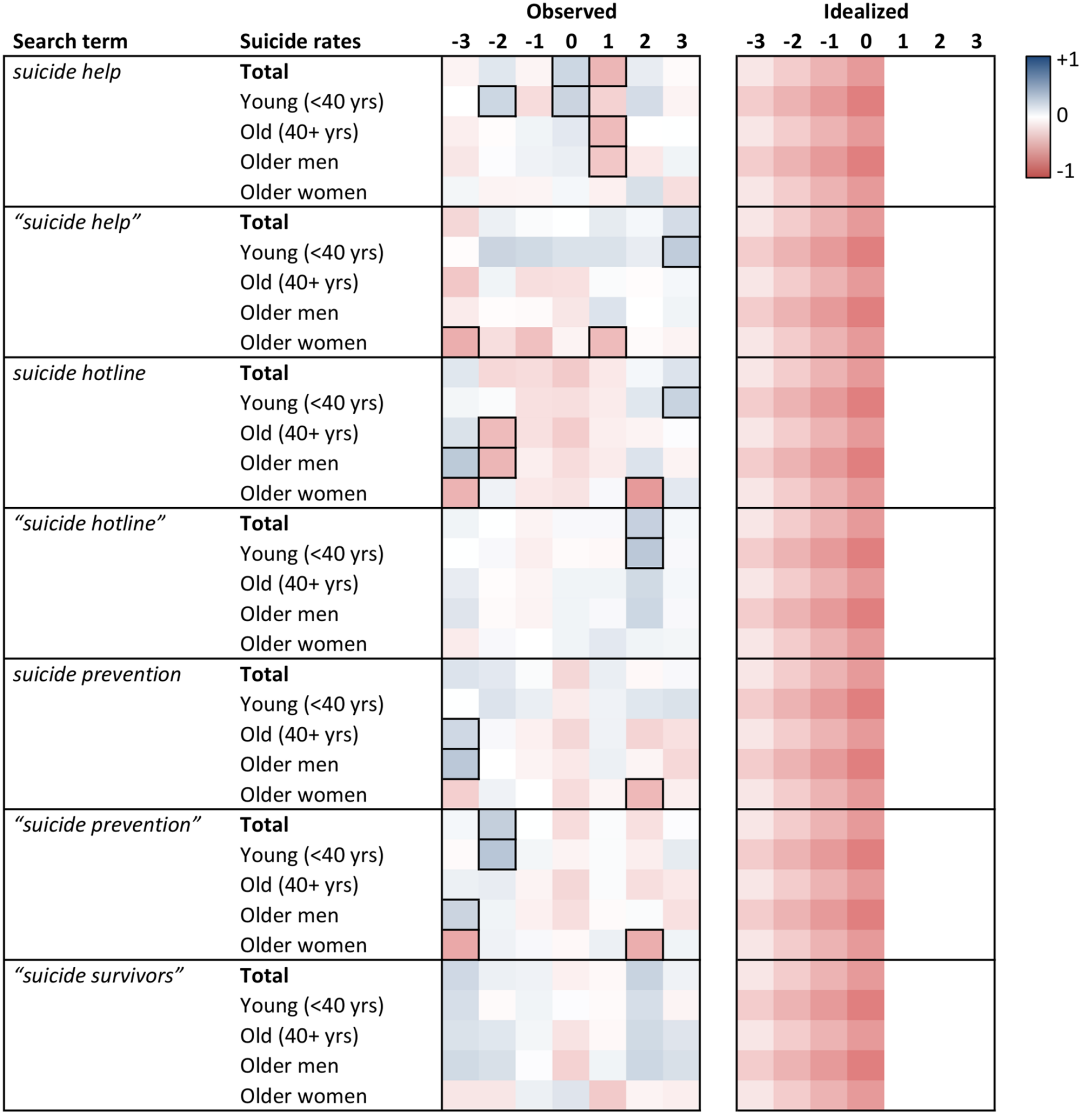
Heat map of cross-correlation coefficients for the US data (suicide prevention terms). Numbers in column headers refer to lags in months. Idealized patterns reflect what could be expected if search volumes predicted suicide rates. In the observed patterns, frames highlight statistically significant (*p* < .05) coefficients.

**Fig 4:**
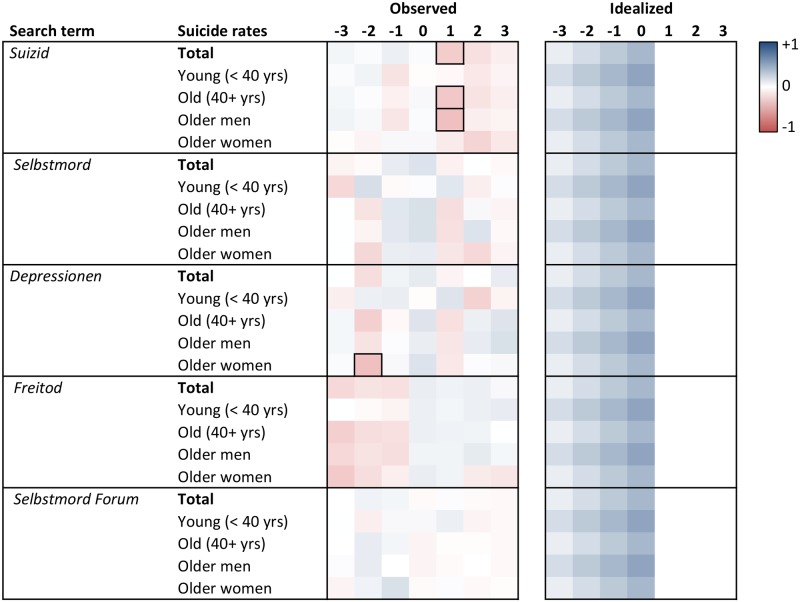
Heat map of cross-correlation coefficients for the German data. *Suizid*, *Selbstmord*, *Freitod* = ‘suicide’ in English; *Depressionen* = ‘depression’ (plural in German); *Selbstmord Forum* = ‘suicide chat’. Numbers in column headers refer to lags in months. Idealized patterns reflect what could be expected if search volumes predicted suicide rates. In the observed patterns, frames highlight statistically significant (*p* < .05) coefficients.

**Fig 5:**
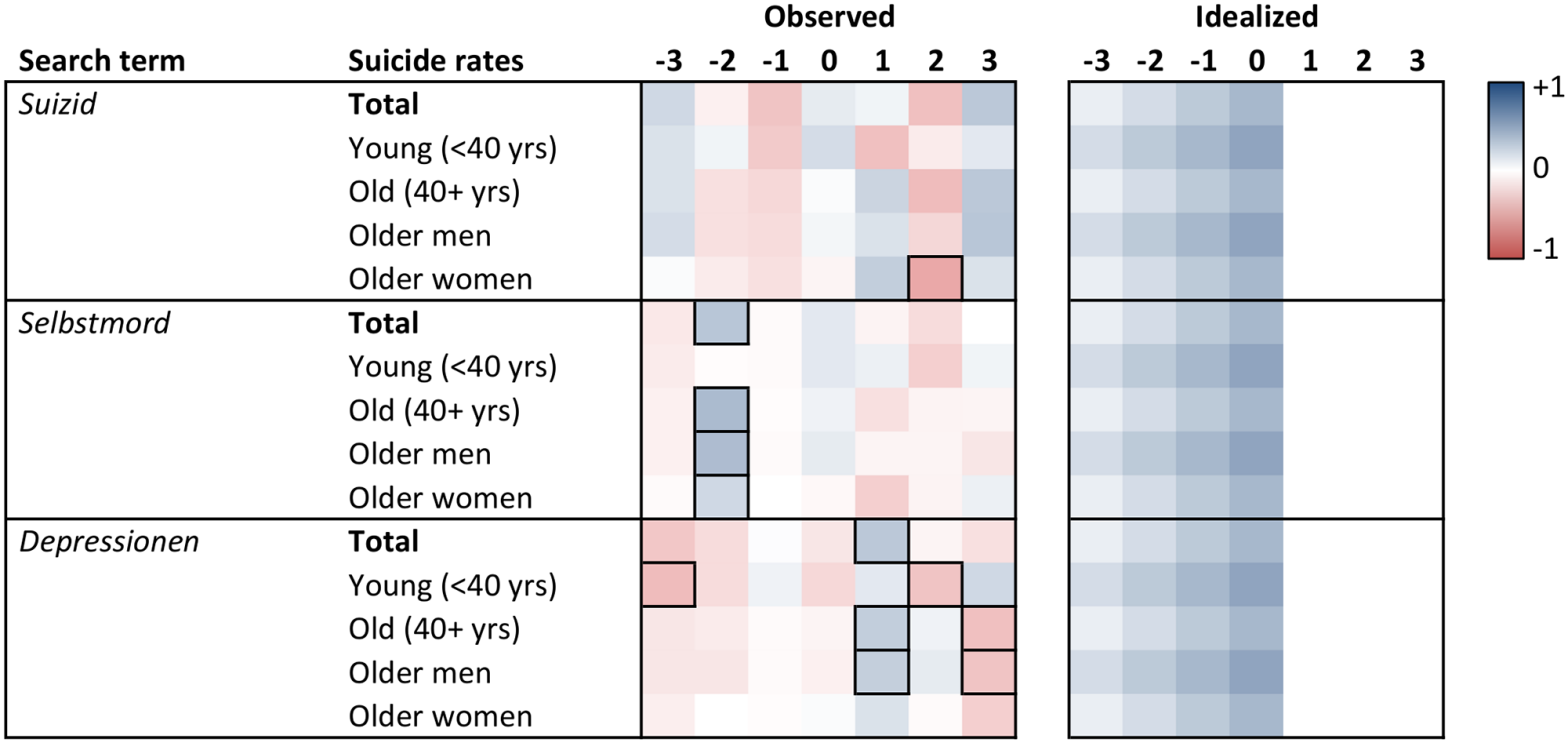
Heat map of cross-correlation coefficients for the Austrian data. *Suizid*, *Selbstmord* = ‘suicide’ in English; *Depressionen* = ‘depression’ (plural in German). Numbers in column headers refer to lags in months. Idealized patterns reflect what could be expected if search volumes predicted suicide rates. In the observed patterns, frames highlight statistically significant (*p* < .05) coefficients.

**Fig 6:**
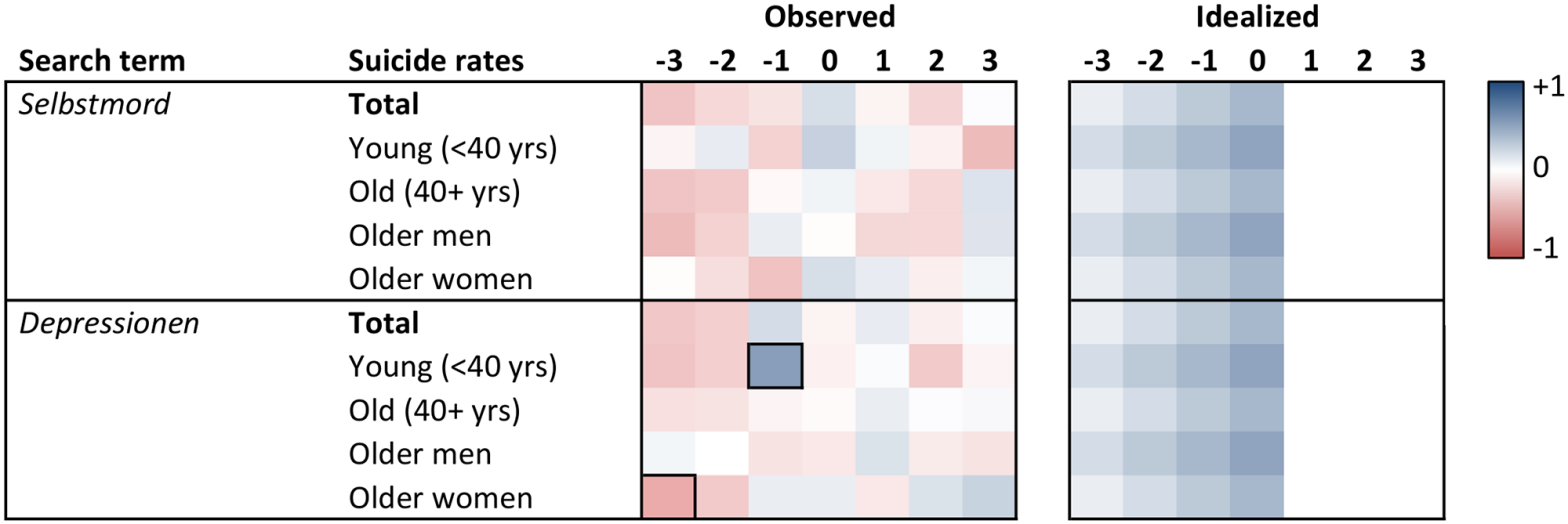
Heat map of cross-correlation coefficients in the Swiss data. *Selbstmord* = ‘suicide’ in English; *Depressionen* = ‘depression’ (plural in German). Numbers in column headers refer to lags in months. Idealized patterns reflect what could be expected if search volumes predicted suicide rates. In the observed patterns, frames highlight statistically significant (*p* < .05) coefficients.

Heat maps of the cross-correlation coefficients of the prewhitened search volumes and suicide rates for lags -3 to +3 are displayed in Figs 1 to 6. Numerical values of the cross-correlation coefficients are provided in S1–S4 Tables. Associations are presented per country and separately for total suicide rates, suicide rates among younger (aged <40 years) and older individuals (aged 40+ years), and among older men and older women. For search volumes of *how to overdose* and *“how to overdose”* in the US data, associations with overall suicide rates of men and women are presented as well. Figs 1 to 6 provide also idealized heat maps that allow for direct visual comparisons of observed and expected cross-correlational patterns.

Overall, associations of the search volumes with suicide rates, irrespective of their statistical significance, were rather weak (*Md* of absolute values = .09, interquartile range = .04 to .17, maximum = .56). Of the 1043 cross-correlation coefficients depicted in Figs 1–6 only 4 (0.38%) indicated large effects (|*r|* ≥ .50), 49 (4.70%) medium-sized effects (.30 ≤ |*r*| < .50), 462 (44.30%) small effects (.10 ≤ |*r*| < .30); the remaining 528 (50.62%) coefficients were in absolute value < .10.

The total number of significant cross-correlation coefficients (8.34%; USA: 9.96%; Germany: 2.29%; Austria: 11.43%; Switzerland: 2.86%) roughly matched the number of expected (5%) Type I errors. Moreover, significant coefficients mostly appeared to lack systematic patterns, both with regards to their expected direction (idealized heat maps), but also with regards to between-country comparisons (compare the patterns for *suicide* in the US data and for *Suizid* or *Selbstmord* in the data of the German-speaking countries, or for *depression* and *Depressionen*, Figs 1 and 4–6) and within-country comparisons (compare the results of search queries with and without quotation marks in the US data, Figs 1–3, or for *Suizid* and *Selbstmord* in the data of the German-speaking countries, Figs 4–6). Associations in the same time-series data also changed direction in adjacent lags, not including lag 0 (observable in the US data for *“how to kill yourself”*, *“how to overdose”*, *online suicide*, and *suicide hotline*, Figs 1 and 2). The two largest and most salient cross-correlation coefficients were found for the US suicide rates of older women and the *online suicide* search volumes. These coefficients were in adjacent lags, but, intriguingly so, had opposing signs (Fig 1): *r* = .56 at lag -3, whereas *r* = -.51 at lag -2. Search volumes of *how to overdose* and *“how to overdose”* had significant cross-correlations with the suicide rates of women; however, patterns of association did not match across search terms with and without quotation marks and changed signs at lag -3 as well (Fig 1).

Patterns appeared somewhat similar for search volumes of *depression* in the USA and *Depressionen* in Germany and Switzerland, insofar as suicide rates of older women in these countries were negatively associated with the respective search volumes at lags -2 and -3 (Figs 1, 4 and 6). Moreover, cross-correlational patterns in the US data for *suicide methods* and *“suicide methods”*, and for *suicide prevention* and *“suicide prevention”*, appeared partly similar to one another (Fig 3). Search volumes of *suicide methods* and “*suicide methods”* were negatively associated with the suicide rates of especially older individuals and older men at lag +1. Search volumes of *suicide prevention* and *“suicide prevention”* showed positive associations with the suicide rates of older men at lag -3, and negative associations with the suicide rates of older women at lag +2.

US search volumes for *suicide* and *how to kill yourself* did not show any associations with suicide rates, but *“how to kill yourself”* did, predominantly at lag -2 (Fig 1; cf. [14]). There were no apparent associations of search volumes of *Selbstmord* with suicide rates in Germany (Fig 4) or Switzerland (Fig 6), but positive associations with suicide rates at lag -2 in Austria (Fig 5). Search volumes of *Suizid* showed associations with suicide rates in Germany (Fig 4) and Austria (Fig 5), but patterns differed widely across these countries.

#### Averaged time-series data

Significant cross-correlation coefficients comparing individual and averaged time series are provided in Table 5. Patterns of association were comparable for US search volumes of *suicide* and *depression*. While there were consistent null findings for *suicide*, observed patterns for *depression* closely resembled one another. Results were different for the German, Austrian, and Swiss data, where reliabilities of individual time series were comparably lower than for the US data (Table 3). In the German data, analyses of individual and averaged time series led to consistent null findings for the search term with the highest reliability, *Selbstmord*. In contrast, patterns of *Suizid* and *Depressionen* differed for individual and averaged time-series data (Table 5). Similarly, patterns did not match for individual and averaged time-series data of *Suizid*, *Selbstmord*, and *Depressionen* in Austria, and of *Depressionen* in Switzerland. Taken together, these results suggest that the unreliability of Google Trends search volumes affect the temporal associations of these data with suicide rates.

**Table 5:** Significant cross-correlation coefficients of individual and averaged time-series data.

Country	Search term	Lag	Individual time-series data	Averaged time-series data
USA^a^	*depression*	-3	-.35** (older men)	-.34** (older men)
		-1	-.23* (younger indiv.)	-.24* (younger indiv.)
		+2	-.24* (older women)	-.23* (older women)
Germany^b^	*Suizid*	+1	-.20* (total)	-.21* (older women)
			-.22* (older indiv.)	
			-.25** (older men)	
		+2		.21* (older men)
	*Depressionen*	-2	-.25** (older women)	
		0		.23* (total)
				.20* (older indiv.)
Austria	*Suizid*	+2	-.34* (older women)	
	*Selbstmord*	-3		-.34** (total)
				-.34** (older indiv.)
				-.33** (older men)
		-2	.32** (total)	
			.38** (older indiv.)	
			.37** (older men)	
			.22* (older women)	
		+3		-.26** (older women)
	*Depressionen*	-3	-.26* (younger indiv.)	
		+1	.31** (total)	
			.28* (older indiv.)	
			.27* (older men)	
		+2	-.23* (younger indiv.)	
		+3	-.25* (older Indiv.)	
			-.23* (older men)	
Switzerland	*Depressionen*	-3	-.33* (older women)	
		-2		-.32* (total)
				-.35* (older indiv.)
		-1	.54** (younger indiv.)	

Numbers are cross-correlation coefficients with overall suicide rates (total) or with suicide rates of younger individuals (<40 years of age), older individuals (40+ years of age), of older men or of older women. Only significant (*p* < .05) cross-correlation coefficients are displayed.

* *p* < .05,

** *p* < .01.

^a,b^ Results for ^a^*suicide*/^b^*Selbstmord* are omitted as cross-correlations failed to reach significance in the respective individual and averaged time-series data.

## Discussion

This study had 3 goals: (1) to systematically collect relevant search terms in the field of suicide research and to assess Google Trends time-series data for availability and suitability for time-series analysis; (2) to conduct rigorous analyses on the temporal associations of search volumes and suicide rates; (3) to compare results for data from USA, Germany, Austria, and Switzerland. This is to date the first and only study in this field of research which directly compared results for different countries.

We found that the number of suitable search query terms, and the reliability of the respective search volume data, increases with country size. Suicide prevention terms could only be investigated for the largest country, the USA. We obtained evidence for temporal associations of Google Trends search volumes with suicide rates, seemingly corroborating previous positive results [12–20]. However, our analyses suggest that most of these associations likely are spurious and lack validity. Patterns differed across countries for comparable search terms and within countries for the same search terms, dependent on the use of quotation marks; for several search terms associations changed their direction in adjacent lags. No plausible psychological mechanisms appear to be at hand for accounting for these peculiarities in the data. Finally, the unreliability of Google Trends search volumes was observed to affect the results for smaller countries. We thus conclude that Google Trends does not appear to be a valid tool for behavioral forecasting of suicidal behavior and that the seemingly confirmative results of previous studies in this area [12–20] need to be interpreted with caution.

One notable result of our study is that search volumes of the probably most salient suicide-related term, *suicide*, failed to show associations with suicide rates in our US data, which conflicts one previous US study [14], but corroborates a similar negative finding of a more recent study [12]. In previous research, *suicide* search volumes also showed no associations with suicide rates in data from the UK [16] and Japan [18] (cf. [15,20]). These findings appear to suggest that salient suicide-related search terms, like *suicide*, are not suited for behavioral forecasting of suicide rates. We also found that alternative terms for *suicide*, like *Selbstmord* in German, may be more relevant or salient search terms for languages other than English. For Switzerland, Google Trends provided no suitable search volume time series for *Suizid* (*suicide*) in the current study; however, data were available for the alternative term *Selbstmord*.

Patterns of associations were inconsistent in the current study and often counterintuitive with regards to the expected direction of association. *Depression* search volumes were mostly negatively associated with suicide rates, especially of older individuals, at various negative time lags in data from the USA, Germany (individual time-series data), Austria (averaged time-series data), and Switzerland; i.e., higher prior search volumes were associated with later lower suicide rates and vice versa (lower search volumes with higher suicide rates). In contrast, *suicide prevention* and *“suicide prevention”* search volumes showed positive associations with the suicide rates of older men at lag -3 in the US data. Contrary to what can be expected, more searches for a term that showed links with higher suicide rates in prior research [15,16,19,20] (*depression*) thus seemingly predicted lower suicide rates, whereas more searches for a suicide prevention term (*suicide prevention*) predicted higher suicide rates. Associations of individual time-series data of the search volume of *Selbstmord* in the Austrian data pointed in the expected, positive direction at negative time lags. However, the reliability of individual time series was found to be low for Austria and Switzerland in the current study and the pattern of association of the respective averaged time series did not match the respective pattern of association of the individual time series.

A recent study [25] suggested that method-specific search requests, like *suicide methods* or *how to hang yourself*, more likely lead to websites with harmful contents than search requests of *suicide*, and that, on average, protective website characteristics outweigh harmful characteristics. We performed an informal Google search for the term *depression* through the Tor browser with an US country ID, which corroborated that the majority of first-ranked websites contained also help pages. Because of actually provided website contents, it appears possible that more requests for terms like *depression* might thus be associated with lower suicide rates. However, more research is needed before any firm conclusions can be drawn from these results. More details need to be known about the contents of websites listed by Google (and other search engines). More needs also to be known about the likelihood of suicidal individuals to actually access and use listed help pages when searching for suicide-related content on the Internet.

Conversely, the positive associations of US suicide prevention search volumes with suicide rates of older men at negative lags appear to imply that these requests increased suicide rates for older men. In the USA, a majority of crisis helpline callers are female [76]. The majority of suicides are by men, thus there appears to be a gender gap in the utilization of crisis helplines. However, if the above offered explanation is applicable (i.e., requests for suicide-related terms prompt help pages that are accessed by users which in turn lowers suicide rates), the positive associations of suicide prevention terms with suicide rates are puzzling. We therefore suggest to interpret these conflicting findings with regards to *depression* and *suicide prevention* as a further indication of spurious results and a likely consequence of the low validity of Google Trends data for behavioral forecasting.

The apparent low validity of Google Trends data for the prediction of suicide rates appears to derive (at least partly so) from a double uncertainty of who searches for suicide-related content on the Internet and why. A recent study [77] reported that 22.5% of young adults in the UK use the Internet to search for content related to suicide or self-harm. While such a use is more prevalent among individuals with suicidal ideation and suicidal intent, most individuals who access harmful sites also access help sites and, overall, more help sites are accessed than harmful sites. Also, a majority of users who search with suicide-related queries actually access entertainment-related content, rather than pro-suicide websites [78]. In order for Google search volumes to be a useful and valid tool for behavioral forecasting of suicide rates, at least two assumptions need to be satisfied: (1) the population that primarily conducts relevant searches is specific and relatively homogeneous (i.e., consists mostly of individuals with suicidal ideation); (2) specific search behavior is strongly linked with specific outcomes (i.e., suicide-related searches increase suicidal behavior). From what can be concluded from our study, as well as from studies on website content [25] and suicide-related search behavior in the general population [77,78], both assumptions likely are not met.

Lazer et al. [36] remarked that advanced features of search engines, like the autocomplete feature of Google (suggesting completed terms for the first few typed-in letters, matched to previous searches of the current user and the most-searched terms of other users) and Google Suggest (suggesting further search terms that might be of relevance, as used in the current study), may also affect the search behavior of users, who are thus nudged to request searches for other terms than originally intended. More general, it currently remains unclear how users actually perform their searches and what factors influence this behavior and to what extent. As for one example, the current study suggests that search volumes of search terms with and without quotations marks differ. According to Google Trends [27] terms with quotation marks lead to more specific results. However, Google Trends presents only normalized search volumes. Therefore, search volumes themselves do not indicate which alternative, with or without quotation marks, is used more frequently. Overall, in the current study more search volumes were available for search terms without quotation marks. We therefore conclude that individuals perform searches typically without quotation marks. Still, more research on the actual search behavior of suicidal individuals, and of the factors influencing this behavior, is needed.

Currently, little is known about the quality of the data gathered from, and made available through, Google Trends, and the algorithm used remains unknown. This study suggests that Google Trends data have severe limitations with regards to their utility for behavioral forecasting of suicide.

### Limitations

Limitations of this study specifically concern the examined Google Trends search volumes and the involved time spans. Individual time-series data were requested on May 2, 2014, data for the averaged time series were requested from April 18, 2014, until April 27, 2014. Investigated time spans covered the years 2004 to 2012. Because of observed inconsistencies in the time-series data, it cannot be ruled out that the same requests taken on other days or months of the year or more recent requests could have led to different results. Analysis of different, or more recent, time spans may also lead to different results as could the analysis of time lags outside the range of -3 to +3 months prior and after suicide events which were considered relevant in the current study. Also, results apply only to a monthly resolution of search volume data and suicide rates. Analyses using a weekly resolution might yield different results.

Analyses of the associations of search volumes with suicide rates of specific age groups and of men and women could not draw on search volumes that were differentiated by age or sex. Google Trends only provides overall normalized search volumes and does not allow to differentiate search volumes by user demographics. Using a cutoff of 40+ years to separate younger and older suicides in analysis may have impacted on results, and differs from some previous studies (e.g., [14]).

Statistical power of significance tests is lower for shorter time series than for longer ones. Some of the time series in the current study contained less than the recommended 50 data points [60]. This may have impacted on results.

Migrants and minorities may perform search requests in other languages than those utilized in the present study for the different countries. The number of language minorities in a country might affect the likelihood of bias in Google Trends data [12]. This issue may be particularly relevant for the Swiss data, but may have had an overall impact on obtained results.

Isolated effects of media reports on suicide (such as coverage of celebrity suicides) on the search volumes and/or the suicide rates were not explicitly addressed in the current study. However, outliers in search volumes that have been caused by media reports were fully accounted for in the modeling of the explanatory time-series data and hence are not expected to have biased obtained results to an undue extent. Still, the potential association of specific large-scale media events with suicide rates warrants additional research.

Access to the Internet varies across, but also within, countries. Access to online media is often diminished in rural areas [79,80]. At the same time, suicide rates may be higher in rural than in urban areas, which, for example, is the case in Austria [81]. Possible differences of specific regions within countries in the temporal associations of search volumes and suicide rates were not considered in the current study.

### Conclusion and perspective

In the present study, data provided by Google Trends appeared to lack reliability and stability over time, and Google Trends data likely are too unspecific to be of use for general behavioral forecasting of suicidal behavior. Future improvements of the algorithm used by Google may improve the quality of data. This may increase its reliability and stability. Direct collaborations of Google with researchers might ultimately increase the transparency, replicability and usefulness of research with Google Trends search volume data (see [36]). For the time being, we recommend requesting Google Trends search volumes repeatedly for the same term(s) on different days and to use their average for research purposes, especially if countries for which data are requested are small. This will at least increase the reliability and stability of data obtained. Search volumes should be requested for terms with and without quotation marks, and the terms should be specific (e.g., *how to hang yourself*), rather than broad (e.g., *suicide*). Adequate methods of data analysis need be used to rule out spurious associations due to merely structural similarities between time series. Finally, more research on the actual search behavior of suicidal individuals, and of the factors influencing this behavior, is needed to gain a deeper insight into the specificity of suicide-related search volumes for national suicide rates.

## Supporting information

S1 Appendix Initial list of search terms. (DOCX)

S2 Appendix Revised list of search terms. (DOCX)

S3 Appendix Search terms suggested by Google Trends. (DOCX)

S4 Appendix Excluded search terms by time series category. (DOCX)

S1 Table Cross-correlations of selected search terms and suicide rates at lags (in months) -3 to +3 in the US data. (DOCX)

S2 Table Cross-correlations of selected search terms and suicide rates at lags (in months) -3 to +3 in the German data. (DOCX)

S3 Table Cross-correlations of selected search terms and suicide rates at lags (in months) -3 to +3 in the Austrian data. (DOCX)

S4 Table Cross-correlations of selected search terms and suicide rates at lags (in months) -3 to +3 in the Swiss data. (DOCX)
