# Supplementary material for: Low validity of Google Trends for behavioral forecasting of national suicide rates
Source: PLoS One. 2017 Aug 16;12(8):e0183149. doi: 10.1371/journal.pone.0183149 (PMC5558943; doi:10.1371/journal.pone.0183149)
Supplement: S1 Appendix — (DOCX) [file pone.0183149.s001.docx]

S1 Appendix. Initial list of search terms.

**English search terms**

Pro-suicide: a suicide, bulletin board system (BBS) on suicide, commit suicide, how to commit suicide, how to kill myself, how to kill yourself, how to suicide, method(s) suicide, rates suicide, sites on suicide, suicidal tendencies, suicide how to, suicide method(s), suicide pact, suicide rates, suicide test, suicide United States of America, suicide USA, teen suicide, test suicide, ways to kill yourself

Suicide prevention: suicidal ideation, suicidal thoughts, suicidal thoughts lyrics, suicide help, suicide prevention

Addition: depression

**German search terms**

Pro-suicide**:** Anleitung Selbstmord, Anleitung Suizid, Arten sich das Leben zu nehmen, Arten sich umzubringen, Arten sich zu töten, Chat Selbstmord, Chat Suizid, Erfahrung Selbstmord, Erfahrung Suizid, Filme Selbstmord, Filme Suizid, Forum Selbstmord, Forum Suizid, Freitod, Gedichte Selbstmord, Gedichte Suizid, Methode(n) Selbstmord, Methode(n) Suizid, mich schmerzfrei umbringen, mich töten. mich schmerzfrei töten, mich umbringen, schmerzlos Selbstmord, schmerzlos Suizid, schnell Selbstmord, schnell Suizid, Selbstmord, Selbstmord *COUNTRY*, Selbstmord Anleitung, Selbstmordanleitung, Selbstmordattentäter, Selbstmord bei Kindern, Selbstmord Chat, Selbstmordchat, Selbstmord Erfahrung, Selbstmorderfahrung, Selbstmordfilme, Selbstmord Filme, Selbstmordforum, Selbstmord Forum, Selbstmord Gedichte, Selbstmordgedichte, Selbstmord Methode(n), Selbstmordmethode(n), Selbstmordrate *COUNTRY*, Selbstmord schmerzlos, Selbstmord schnell, Selbstmord Sprüche, Selbstmordsprüche, Selbstmordstatistik *COUNTRY*, Selbstmord Statistik *COUNTRY*, Selbstmord strafbar, Selbsttötung, Sprüche Selbstmord, Sprüche Suizid Suizidsprüche, Statistik Selbstmord *COUNTRY*, Statistik Suizid *COUNTRY*, strafbar Selbstmord, strafbar Suizid, Suizid, Suizid *COUNTRY*, Suizidstatistik *COUNTRY*, Suizidalität, Suizid Anleitung, Suizidanleitung, Suizid bei Kindern, Suizidchat, Suizid Chat, Suiziderfahrung, Suizid Erfahrung, Suizid Filme, Suizidfilme, Suizidforum, Suizid Forum, Suizid Gedichte, Suizidgedichte, Suizid Methode(n), Suizidmethode(n), Suizidrate *COUNTRY*, Suizid schmerzlos, Suizid schnell, Suizid Sprüche, Suizid Statistik *COUNTRY*, Suizid strafbar, Wege sich das Leben zu nehmen, Wege sich umzubringen, Wege sich zu töten

*COUNTRY* = Deutschland, Österreich or Schweiz

Suicide prevention: Hilfe Selbstmord, Hilfe Suizid, Hotline Selbstmord, Hotline Suizid, Prävention Selbstmord, Prävention Suizid, Selbstmordgedanken, Selbstmord Hilfe, Selbstmordhilfe, Selbstmordhotline, Selbstmord Hotline, Selbstmord Prävention, Selbstmordprävention, Selbstmord Test, Selbstmordtest, Selbstmord vorbeugen, Suizidgedanken, Suizid Hilfe, Suizidhilfe, Suizid Hotline, Suizidhotline, Suizid Prävention, Suizidprävention, Suizidtest, Suizid Test, Suizid vorbeugen, Test Selbstmord, Test Suizid, vorbeugen Selbstmord, vorbeugen Suizid

Addition: Depression
