# Supplementary material for: Low validity of Google Trends for behavioral forecasting of national suicide rates
Source: PLoS One. 2017 Aug 16;12(8):e0183149. doi: 10.1371/journal.pone.0183149 (PMC5558943; doi:10.1371/journal.pone.0183149)
Supplement: S2 Appendix — (DOCX) [file pone.0183149.s002.docx]

S2 Appendix. Revised list of search terms.

**English search terms**

Pro-suicide: attempted suicide, bulletin board system (BBS) on suicide, chat suicide, child suicide, commit suicide, fast suicide, forum suicide, guide suicide, how to commit suicide, how to kill yourself, how to suicide, I don’t want to live, instruction suicide, I want to die, manual suicide, method(s) suicide, painless suicide, quick suicide, sites on suicide, suicidality, suicide, suicide attempt, suicide chat, suicide child, suicide fast, suicide forum, suicide guide, suicide how to, suicide instruction, suicide manual, suicide method(s), suicide painless, suicide partner, suicide quick

Prevention: bereaved suicide, bereavement suicide, help suicide, hotline suicide , prevention suicide, reason(s) suicide, suicidal ideation, suicidal thoughts, suicide bereavement, suicide help, suicide hotline, suicide prevention, suicide reason(s), suicide support group, suicide thoughts, support group suicide

Methods: how to hang yourself, how to poison yourself, how to shoot yourself

Addition: depression

**German search terms**

Pro-suicide: Anleitung Selbstmord, Anleitung Suizid, Arten sich das Leben zu nehmen, Arten sich umzubringen, Chat Selbstmord, Chat Suizid, Forum Selbstmord, Forum Suizid, Freitod, Ich möchte nicht mehr leben, Ich will nicht mehr leben, Ich will sterben, Kinder Selbstmord, Kinder Suizid, Methode(n) Selbstmord, Methode(n) Suizid, Partner Selbstmord, Partnerselbstmord, Partner Suizid, Partnersuizid, schmerzlos(er) Selbstmord, schmerzlos(er) Suizid, schnell(er) Selbstmord, schnell(er) Suizid, Seite Selbstmord, Seite Suizid, Selbstmord, Selbstmord (bei) Kinder(n), Selbstmordseite, Selbstmord Anleitung, Selbstmordanleitung, Selbstmord Chat, Selbstmordchat, Selbstmord Forum, Selbstmordforum, Selbstmordmethode(n), Selbstmord Methode(n), Selbstmordpartner, Selbstmord Partner, Selbstmord schmerzlos, Selbstmord schnell, Selbstmord Seite, Selbstmordversuch, sich schmerzfrei umbringen, sich umbringen, Suizid, Suizid (bei) Kinder(n), Suizidalität, Suizid Anleitung, Suizidanleitung, Suizid Chat, Suizidchat, Suizid Forum, Suizidforum, Suizid Methode(n), Suizidmethode(n), Suizid Partner, Suizidpartner, Suizid schmerzlos, Suizid schnell, Suizid Seite, Suizidseite, wie bringe ich mich um, wie erhänge ich mich, wie erschieße ich mich, wie vergifte ich mich

Suicide prevention: Grund Selbstmord, Grund Suizid, Gründe Selbstmord, Gründe Suizid, Hilfe Selbstmord, Hilfe Suizid, Hotline Selbstmord, Hotline Suizid, Ich will leben, Selbsthilfegruppe Selbstmord, Selbsthilfegruppe Suizid, Selbstmordgedanken, Selbstmord Grund, Selbstmordgrund, Selbstmordgründe, Selbstmord Gründe, Selbstmord Hilfe, Selbstmordhilfe, Selbstmord Hotline, Selbstmordhotline, Selbstmord Selbsthilfegruppe, Selbstmordselbsthilfegruppe, Selbstmord Trauer, Suizidgedanken, Suizidgrund, Suizid Grund, Suizid Gründe, Suizidgründe, Suizid Hilfe, Suizidhilfe, Suizid Hotline, Suizidhotline, Suizid Selbsthilfegruppe, Suizidselbsthilfegruppe, Suizid Trauer, Trauer Selbstmord, Trauer Suizid

Addition: Depression, Depressionen
