# Supplementary material for: Low validity of Google Trends for behavioral forecasting of national suicide rates
Source: PLoS One. 2017 Aug 16;12(8):e0183149. doi: 10.1371/journal.pone.0183149 (PMC5558943; doi:10.1371/journal.pone.0183149)
Supplement: S3 Appendix — (DOCX) [file pone.0183149.s003.docx]

**S3 Appendix. Search terms suggested by Google Trends.**

**USA**

after attempted suicide, after suicide attempt, American suicide prevention, army suicide, army suicide prevention, army suicides, attempt to suicide, Bay bridge suicides, best friend suicide, best suicide, best suicide methods, best ways suicide, best way suicide, bipolar suicidal ideation, bridge jump suicide, bridge suicides, bullying suicides, carbon monoxide suicide, causes for suicide, causes of suicide, chat room suicide, child abuse suicide, child commits suicide, child suicidal, child suicidal thoughts, child suicide prevention, child support suicide, commit a suicide, commit suicide fast, commit suicide painless, commit suicide pills, complete suicide manual, crisis hotline, cyberbullying suicides, cyber bullying suicides, depressions, depression suicidal ideation, depression suicidal thoughts, depression suicide help, drug overdose suicide, easiest suicide, easiest suicide method, easiest ways suicide, easy fast suicide, easy painless suicide, easy suicide, easy suicide methods, easy suicide painless, easy suicide ways, failed suicide attempt, fast easy suicide, fast painless suicide, forums suicide, golden gate suicide, golden gate suicides, guide to suicide, hanging yourself, hanging yourself suicide, hang yourself, helium and suicide, helium for suicide, helium suicide, helium suicide method, helium tank suicide, help for suicidal, help for suicide, help with suicide, hotline for suicide, how commit suicide, how to commit suicide painlessly, how to die, I attempted suicide, I kill myself, I wanna die, I wanna kill myself, I’m killing myself, jump off bridge, killing myself, killing yourself, kill myself, kill yourself, kill your self, kill yourself fast, kill yourself painlessly, live suicide, live suicide video, method of suicide, methods for suicide, methods of suicide, military and suicide, military suicide, military suicide prevention, military suicides, most painless suicide, national suicide hotline, national suicide prevention, online suicide chat, online suicide help, online suicide hotline, online suicide prevention, overdose sleeping pills, overdose suicide, overdose suicide methods, painless fast suicide, painless methods suicide, painless quick suicide, painless suicide method, painless suicide methods, painless suicide ways, painless ways suicide, partner suicide, passive suicidal ideation, pills for suicide, prevention of suicide, quick easy suicide, quick painless suicide, quick suicide methods, reason for suicide, reasons for suicide, reasons of suicide, reasons to suicide, signs of suicidal, signs of suicide, sites suicide, site suicide, skyway bridge suicides, sleeping pills suicide, sleeping pill suicide, suicidal, suicidal depression, suicidal help, suicidal ideation treatment, suicidal signs, suicidal symptoms, suicidal thoughts help, suicidal warning signs, suicide attempt survivors, suicide bag, suicide bag helium, suicide best method, suicide best methods, suicide best ways, suicide bridge, suicide by hanging, suicide by helium, suicide by method, suicide by overdose, suicide by pills, suicide chat hotline, suicide chat online, suicide chat room, suicide chat rooms, suicide crisis hotline, suicide easiest way, suicide easy, suicide easy methods, suicide easy ways, suicide exit bag, suicide family survivors, suicide forums, suicide from bridge, suicide from bullying, suicide groups, suicide hanging, suicide help chat, suicide help hotline, suicide help online, suicide hotline chat, suicide hotline help, suicide hotline number, suicide hotline online, suicide ideation, suicide in army, suicide in military, suicide in youth, suicide is easy, suicide kit, suicide kit helium, suicide kits, suicide methods hanging, suicide of child, suicide off bridge, suicide of reason, suicide on bridge, suicide online chat, suicide on video, suicide overdose methods, suicide overdose pills, suicide painless methods, suicide plastic bag, suicide prevention army, suicide prevention center, suicide prevention chat, suicide prevention day, suicide prevention hotline, suicide prevention lifeline, suicide prevention training, suicide prevention walk, suicide quick painless, suicides, suicides and bullying, suicides by bullying, suicides from bullying, suicides in army, suicide site, suicide sites, suicide sleeping pills, suicide support, suicide support groups, suicide survivors forum, suicide survivors group, suicide survivors support, suicide video, suicide videos, suicide warning signs, suicide ways, suicide with helium, suicide with pills, support for suicide, survivors of suicide, symptoms of suicide, teenage suicide, teenage suicide depression, teenage suicide reasons, teenage suicides, the suicide hotline, the suicide manual, thoughts about suicide, thoughts of suicidal, thoughts of suicide, thoughts on suicide, to kill yourself, top suicide reasons, to suicide, to suicide attempt, video of suicide, video on suicide, wanna kill myself, ways of suicide, ways to die, ways to kill yourself, ways to overdose, why commit suicide, youth and suicide, youth suicide, youth suicide prevention

**Germany**

Anleitung zum Selbstmord, beste Selbstmordmethode, Depression Selbstmord, Depression Suizid, Depression und Suizid, Facebook Selbstmord, Facebook Suizid, Freitod Forum, Freitod Methoden, Freitod wie, Gründe für Selbstmord, Hilfe bei Selbstmord, Hilfe bei Selbstmordgedanken, Hilfe bei Suizid, Hilfe Selbstmordgedanken, Hilfe Suizidgedanken, Ich möchte leben, Ich möchte sterben, mit Tabletten Selbstmord, mit Tabletten Suizid, nach Selbstmordversuch, Schlaftabletten Selbstmord, Schlaftabletten Suizid, Selbstmord aber wie, Selbstmord begehen, Selbstmord begehen wie, Selbstmord Depression, Selbstmord durch Tabletten, Selbstmord Facebook, Selbstmordgedanken Depression, Selbstmordgedanken Hilfe, Selbstmordgedanken was tun, Selbstmordmethoden schmerzlos, Selbstmord mit Schlaftabletten, Selbstmord mit Tabletten, Selbstmord Schlaftabletten, Selbstmord schmerzlos schnell, Selbstmord schnell schmerzlos, Selbstmord Tabletten, Selbstmord Tipps, Selbstmord Tipps schmerzlos, Selbstmord wegen Facebook, Selbstmord welche Tabletten, Selbstmord wie, Selbstmord Zug, sich selbst umbringen, sich umbringen wie, Suizid Depression, Suizid Facebook, Suizid Forum Methoden, Suizidgedanken was tun, Suizid mit Tabletten, Suizid Schlaftabletten, Suizid Tabletten, Suizid Tipps, Suizid wie, Suizid Zug, Tabletten für Selbstmord, Tabletten Selbstmord, Tabletten Suizid, Tipps Selbstmord, Tipps Suizid, wie geht Selbstmord, wie Selbstmord, wie Selbstmord begehen, wie Suizid, Zug Selbstmord, Zug Suizid, Überdosis Schlaftabletten

**Austria**

Anleitung zum Selbstmord, Facebook Selbstmord, Selbstmord Facebook, Selbstmord wie, Selbstmord Zug, Suizid wie, wie Selbstmord, Zug Selbstmord

**Switzerland**

Selbstmord wie, Selbstmord Zug, Suizid Zug, wie Selbstmord, Zug Selbstmord, Zug

Suizid
