# Supplementary material for: Low validity of Google Trends for behavioral forecasting of national suicide rates
Source: PLoS One. 2017 Aug 16;12(8):e0183149. doi: 10.1371/journal.pone.0183149 (PMC5558943; doi:10.1371/journal.pone.0183149)
Supplement: S4 Appendix — (DOCX) [file pone.0183149.s004.docx]

**S4 Appendix. Excluded search terms by time series category.**

**USA**

Complete times series

best suicide, causes of suicide, chat suicide, child suicide, commit suicide, depressions, help suicide, hotline suicide, how commit suicide, how to commit suicide, how to die, how to suicide, I kill myself, I wanna die, I want to die, kill myself, kill yourself, methods suicide, overdose suicide, prevention suicide, reasons suicide, suicidal, suicide attempt, suicide bridge, suicide child, suicide groups, suicide how to, suicide painless, suicides, suicide support, suicide video, suicide ways, teenage suicide, to kill yourself, to suicide, ways to die, ways to kill yourself, youth suicide

Incomplete times series

American suicide prevention, army suicide, army suicide prevention, attempted suicide, attempt to suicide, best ways suicide, best way suicide, bridge suicides, carbon monoxide suicide, causes for suicide, child abuse suicide, commit a suicide, commit suicide painless, commit suicide pills, crisis hotline, depression suicidal thoughts, depression suicide help, easiest suicide, easy suicide, easy suicide ways, fast suicide, forums suicide, forum suicide, golden gate suicide, golden gate suicides, guide suicide, guide to suicide, hanging yourself, hang yourself, helium suicide, help for suicidal, help for suicide, help with suicide, hotline for suicide, how to hang yourself, I don’t want to live, jump off bridge, killing myself, killing yourself, kill your self, kill yourself painlessly, live suicide, method of suicide, methods of suicide, method suicide, military suicide, military suicides, national suicide hotline, national suicide prevention, online suicide help, overdose sleeping pills, painless methods suicide, painless suicide methods, painless suicide ways, painless ways suicide, prevention of suicide, quick suicide, reasons for suicide, reasons of suicide, reasons to suicide, signs of suicidal, signs of suicide, sites suicide, site suicide, sleeping pills suicide, suicidal depression, suicidal help, suicidal ideation, suicidality, suicidal signs, suicidal symptoms, suicidal thoughts, suicidal thoughts help, suicide bag, suicide best ways, suicide by hanging, suicide by overdose, suicide crisis hotline, suicide easiest way, suicide easy, suicide fast, suicide forum, suicide from bullying, suicide guide, suicide hanging, suicide help hotline, suicide hotline help, suicide ideation, suicide in army, suicide in military, suicide in youth, suicide kit, suicide kits, suicide method, suicide of child, suicide off bridge, suicide on bridge, suicide on video, suicide overdose pills, suicide painless methods, suicide prevention army, suicide prevention center, suicide prevention hotline, suicide quick, suicide reason, suicide site, suicide sites, suicide sleeping pills, suicide support groups, suicide thoughts, suicide videos, suicide warning signs, suicide with pills, support for suicide, support group suicide, survivors of suicide, symptoms of suicide, thoughts of suicidal, thoughts of suicide, to suicide attempt, video of suicide, video on suicide, ways of suicide, ways to overdose, why commit suicide, youth and suicide, youth suicide prevention

Single peaks

after attempted suicide, after suicide attempt, army suicides, bereavement suicide, best friend suicide, best suicide methods, bipolar suicidal ideation, bridge jump suicide, bullying suicides, chat room suicide, child commits suicide, child suicidal, child suicidal thoughts, child suicide prevention, child support suicide, commit suicide fast, complete suicide manual, cyberbullying suicides, cyber bullying suicides, depression suicidal ideation, drug overdose suicide, easiest ways suicide, easy painless suicide, easy suicide methods, easy suicide painless, failed suicide attempt, fast painless suicide, hanging yourself suicide, helium for suicide, helium tank suicide, how to commit suicide painlessly, how to poison yourself, how to shoot yourself, I attempted suicide, I wanna kill myself, I’m killing myself, kill yourself fast, live suicide video, manual suicide, methods for suicide, military and suicide, military suicide prevention, most painless suicide, online suicide chat, online suicide hotline, online suicide prevention, overdose suicide methods, painless fast suicide, painless quick suicide, partner suicide, passive suicidal ideation, pills for suicide, quick easy suicide, quick painless suicide, quick suicide methods, reason for suicide, reason suicide, skyway bridge suicides, sleeping pill suicide, suicide hotline number, suicide of reason, suicidal warning signs, suicide bag helium, suicide bereavement, suicide best methods, suicide best method, suicide by helium, suicide by method, suicide by pills, suicide chat hotline, suicide chat online, suicide chat room, suicide chat rooms, suicide easy methods, suicide easy ways, suicide exit bag, suicide forums, suicide from bridge, suicide help chat, suicide help online, suicide hotline chat, suicide hotline online, suicide is easy, suicide kit helium, suicide manual, suicide online chat, suicide overdose methods, suicide partner, suicide plastic bag, suicide prevention chat, suicide prevention day, suicide prevention lifeline, suicide prevention training, suicide prevention walk, suicide quick painless, suicides and bullying, suicides by bullying, suicides from bullying, suicide support group, suicide survivors group, suicide survivors support, suicide with helium, teenage suicide depression, teenage suicides, the suicide hotline, the suicide manual, thoughts about suicide, thoughts on suicide, top suicide reasons, wanna kill myself

Search terms suggested by Google Trends without results

Bay bridge suicides, easiest suicide method, easy fast suicide, fast easy suicide, helium and suicide, helium suicide method, painless suicide method, suicidal ideation treatment, suicide attempt survivors, suicide family survivors, suicide methods hanging, suicides in army, suicide survivors forum, teenage suicide reason

**Germany**

Complete times series

Depression, Ich will leben

Incomplete times series

Chat Suizid, Forum Selbstmord, Forum Suizid, Ich will sterben, Selbstmordforum, Selbstmordgedanken, Selbstmordversuch, Selbstmord wie, Selbstmord Zug, Suizidalität, Suizid Chat, Suizid Forum, wie Selbstmord, Zug Selbstmord

Single peaks

Anleitung Selbstmord, Anleitung Suizid, Chat Selbstmord, Depression Selbstmord, Depression Suizid, Facebook Selbstmord, Facebook Suizid, Freitod Methoden, Gründe Selbstmord, Hilfe Selbstmord, Hilfe Suizid, Hotline Selbstmord, Ich möchte leben, Ich möchte nicht mehr leben, Ich möchte sterben, Ich will nicht mehr leben, Kinder Selbstmord, Kinder Suizid, Methoden Selbstmord, Methoden Suizid, Methode Selbstmord, mit Tabletten Selbstmord, mit Tabletten Suizid, Schlaftabletten Selbstmord, Schlaftabletten Suizid, schmerzloser Selbstmord, schmerzloser Suizid, schneller Selbstmord, schnell Selbstmord, Selbsthilfegruppe Selbstmord, Selbstmord Anleitung, Selbstmord begehen, Selbstmord begehen wie, Selbstmord Chat, Selbstmord Depression, Selbstmord Facebook, Selbstmordgedanken Hilfe, Selbstmordgedanken was tun, Selbstmord Gründe, Selbstmord Hilfe, Selbstmordhilfe, Selbstmord Hotline, Selbstmord Kinder, Selbstmord Methode, Selbstmordmethode, Selbstmord Methoden, Selbstmordmethoden, Selbstmord mit Schlaftabletten, Selbstmord mit Tabletten, Selbstmord Schlaftabletten, Selbstmord schmerzlos, Selbstmord schnell, Selbstmord Tabletten, Selbstmord Tipps, Selbstmord Tipps schmerzlos, Selbstmord Trauer, Selbstmord wegen Facebook, sich umbringen, sich umbringen wie, Suizidanleitung, Suizid Anleitung, Suizidchat, Suizid Depression, Suizid Facebook, Suizidforum, Suizid Forum Methoden, Suizidgedanken, Suizid Hilfe, Suizid Kinder, Suizid Methoden, Suizidmethoden, Suizid mit Tabletten, Suizid Schlaftabletten, Suizid Tabletten, Suizid Tipps, Suizid Trauer, Suizid wie, Suizid Zug, Tabletten Selbstmord, Tabletten Suizid, Tipps Selbstmord, Tipps Suizid, Trauer Selbstmord, Trauer Suizid, wie bringe ich mich um, wie Selbstmord begehen, wie Suizid, Zug Suizid, Überdosis Schlaftabletten

Search terms suggested by Google Trends without results

Anleitung zum Selbstmord, beste Selbstmordmethode, Depression und Suizid, Freitod Forum, Freitod wie, Gründe für Selbstmord, Hilfe bei Selbstmord, Hilfe bei Selbstmordgedanken, Hilfe bei Suizid, Hilfe Selbstmordgedanken, Hilfe Suizidgedanken, nach Selbstmordversuch, Selbstmord aber wie, Selbstmord durch Tabletten, Selbstmordgedanken Depression, Selbstmordmethoden schmerzlos, Selbstmord schmerzlos schnell, Selbstmord schnell schmerzlos, Selbstmord welche Tabletten, sich selbst umbringen, Suizidgedanken was tun, Tabletten für Selbstmord, wie geht Selbstmord

**Austria**

Complete times series

Depression

Incomplete times series

Ich will leben

Single peaks

Anleitung Selbstmord, Anleitung zum Selbstmord, Facebook Selbstmord, Forum Selbstmord, Forum Suizid, Freitod, Ich will sterben, Selbstmord Anleitung, Selbstmord Facebook, Selbstmordgedanken, Selbstmordmethoden, Selbstmordversuch, Selbstmord wie, Selbstmord Zug, Suizid Forum, wie bringe ich mich um, wie Selbstmord, Zug Selbstmord

Search terms suggested by Google Trends without results

Selbstmord Forum, Suizidgedanken, Suizid wie

**Switzerland**

Complete times series

Depression

Single peaks

Forum Suizid, Freitod, Ich will leben, Ich will sterben, Selbstmordmethoden, Selbstmord wie, Selbstmord Zug, Suizid Forum, Suizidgedanken, Suizid Zug, wie bringe ich mich um, wie Selbstmord, Zug Selbstmord

Search terms suggested by Google Trends without results

Zug Suizid
