# Supplementary material for: Low validity of Google Trends for behavioral forecasting of national suicide rates
Source: PLoS One. 2017 Aug 16;12(8):e0183149. doi: 10.1371/journal.pone.0183149 (PMC5558943; doi:10.1371/journal.pone.0183149)
Supplement: S1 Table — (DOCX) [file pone.0183149.s005.docx]

**S1 Table. Cross-correlations of selected search terms and suicide rates at lags (in months) -3 to +3 in the US data.**

|  |  | Lag (in months) | | | | | | |
| --- | --- | --- | --- | --- | --- | --- | --- | --- |
| Search term | Suicide rates | -3 | -2 | -1 | 0 | +1 | +2 | +3 |
| *suicide* | Total | -.08 | .14 | -.09 | .04 | -.04 | .01 | .01 |
|  | Young (<40 yrs) | -.13 | .08 | -.05 | .07 | -.06 | .09 | .05 |
|  | Old (40+ yrs) | .00 | .14 | -.10 | .00 | .00 | -.07 | -.04 |
|  | Older men | .02 | .10 | .02 | -.01 | .07 | -.09 | .01 |
|  | Older women | -.05 | .05 | -.22 | .01 | -.15 | .07 | -.09 |
| *depression* | Total | -.07 | -.01 | -.20 | .04 | .01 | -.13 | .00 |
|  | Young (<40 yrs) | -.01 | .04 | **-.23*** | .14 | .04 | -.10 | .04 |
|  | Old (40+ yrs) | -.10 | -.06 | -.08 | -.08 | -.02 | -.10 | -.05 |
|  | Older men | .08 | -.11 | .01 | -.09 | -.05 | .03 | -.05 |
|  | Older women | **-.35**** | .13 | -.17 | .03 | .06 | **-.24*** | .01 |
| *how to kill yourself* | Total | .09 | -.08 | .12 | -.12 | .03 | .07 | -.07 |

|  | Young (<40 yrs) | .15 | -.14 | .11 | -.14 | .00 | .05 | -.11 |
| --- | --- | --- | --- | --- | --- | --- | --- | --- |
|  | Old (40+ yrs) | .05 | -.04 | .11 | -.10 | .04 | .07 | -.04 |
|  | Older men | .07 | -.03 | .12 | -.10 | .03 | .03 | -.01 |
|  | Older women | -.03 | -.05 | .05 | -.05 | .05 | .20 | -.16 |
| *“how to kill yourself”* | Total | .18 | **-.35**** | -.04 | -.03 | -.22 | .04 | -.17 |
|  | Young (<40 yrs) | **.26*** | **-.36**** | -.04 | .07 | **-.26*** | .08 | -.13 |
|  | Old (40+ yrs) | .12 | **-.32**** | -.04 | -.09 | -.17 | .01 | -.18 |
|  | Older men | .15 | **-.36**** | .01 | -.08 | -.19 | -.01 | -.20 |
|  | Older women | -.01 | -.07 | -.16 | -.09 | -.06 | .06 | -.03 |
| *how to overdose* | Total | .05 | -.17 | .01 | .14 | .09 | .18 | -.01 |
|  | Men | .19 | -.24 | .01 | .21 | .14 | .00 | -.11 |
|  | Women | **-.34**** | .15 | .00 | -.16 | -.14 | **.44**** | .24 |
|  | Young (<40 yrs) | .18 | -.13 | -.04 | .04 | .02 | .01 | .16 |
|  | Old (40+ yrs) | -.10 | -.14 | .04 | .19 | .11 | **.27*** | -.17 |
|  | Older men | -.02 | -.23 | .10 | .**28*** | .11 | .07 | **-.27*** |
|  | Older women | -.14 | .20 | -.12 | -.21 | -.01 | **.37**** | .22 |
| *“how to overdose”* | Total | .02 | .18 | -.03 | -.25 | .31 | .24 | **-.42**** |
|  | Men | -.10 | .19 | .08 | .21 | .28 | .18 | **-.47**** |
|  | Women | **.39*** | -.05 | **-.38*** | .12 | .08 | .17 | .17 |
|  | Young (<40 yrs) | -.17 | **.33*** | .23 | **-.50**** | .15 | **.42**** | **-.42**** |
|  | Old (40+ yrs) | .21 | -.08 | -.30 | .14 | **.34*** | -.07 | -.23 |
|  | Older men | -.01 | .10 | -.08 | .08 | .27 | -.03 | **-.35*** |
|  | Older women | **.42**** | **-.34*** | **-.40*** | .10 | .09 | -.08 | .31 |
| *online suicide* | Total | .05 | .00 | -.09 | .20 | -.25 | **.32*** | -.25 |
|  | Young (<40 yrs) | .28 | -.20 | .13 | -.09 | -.02 | .18 | -.15 |
|  | Old (40+ yrs) | -.21 | .19 | -.27 | **.39**** | **-.37**** | **.32*** | -.24 |
|  | Older men | **-.46**** | **.41**** | -.27 | .22 | -.15 | .15 | -.18 |
|  | Older women | **.56**** | **-.51**** | .09 | .23 | **-.36**** | .26 | -.04 |
| *painless suicide* | Total | .00 | .00 | -.06 | .08 | -.01 | .16 | -.01 |
|  | Young (<40 yrs) | .05 | -.02 | -.07 | .04 | .03 | .13 | -.03 |
|  | Old (40+ yrs) | -.04 | .01 | -.05 | .10 | -.03 | .16 | .00 |
|  | Older men | -.04 | -.04 | -.01 | .10 | -.06 | .17 | -.02 |
|  | Older women | -.03 | .15 | -.13 | .06 | .10 | .04 | .06 |
| *“painless suicide”* | Total | .12 | -.27 | .20 | -.25 | .09 | -.13 | -.11 |
|  | Young (<40 yrs) | .05 | -.25 | .18 | -.21 | .15 | -.12 | -.18 |
|  | Old (40+ yrs) | .14 | -.24 | .19 | -.25 | .05 | -.12 | -.06 |
|  | Older men | .15 | -.23 | .16 | -.24 | .05 | -.10 | -.10 |
|  | Older women | .05 | -.18 | .19 | -.17 | .03 | -.15 | .08 |
| *“suicide chat”* | Total | .09 | -.16 | .03 | -.21 | -.02 | -.04 | -.17 |
|  | Young (<40 yrs) | .23 | -.07 | .06 | -.21 | .04 | .06 | -.12 |
|  | Old (40+ yrs) | .01 | -.19 | .01 | -.19 | -.05 | -.09 | -.18 |
|  | Older men | .01 | -.18 | .03 | -.17 | -.01 | -.06 | -.18 |
|  | Older women | .00 | -.15 | -.08 | -.22 | -.16 | -.14 | -.13 |
| *suicide methods* | Total | -.18 | .05 | -.17 | -.02 | **-.30**** | .16 | -.13 |
|  | Young (<40 yrs) | -.20 | .05 | -.16 | .02 | **-.26*** | .18 | -.10 |
|  | Old (40+ yrs) | -.15 | .04 | -.15 | -.04 | **-.28**** | .13 | -.13 |
|  | Older men | -.14 | .06 | -.10 | -.05 | **-.29**** | .12 | -.13 |
|  | Older women | -.12 | -.04 | **-.22*** | .01 | -.14 | .11 | -.07 |
| *“suicide methods”* | Total | -.04 | -.08 | -.16 | -.01 | **-.24*** | .13 | -.07 |
|  | Young (<40 yrs) | .00 | -.06 | -.17 | .04 | -.18 | .12 | -.04 |
|  | Old (40+ yrs) | -.06 | -.09 | -.14 | -.04 | **-.25*** | .12 | .08 |
|  | Older men | -.04 | -.07 | -.12 | -.04 | **-.26*** | .14 | -.10 |
|  | Older women | -.10 | -.10 | -.12 | -.04 | -.10 | .00 | .02 |
| *suicide help* | Total | -.05 | .14 | -.04 | **.23*** | **-.29**** | .11 | -.02 |
|  | Young (<40 yrs) | .00 | **.23*** | -.14 | **.24*** | -.18 | .20 | -.05 |
|  | Old (40+ yrs) | -.07 | -.01 | .07 | .13 | **-.27*** | .00 | .01 |
|  | Older men | -.10 | .02 | .08 | .10 | **-.22*** | -.09 | .07 |
|  | Older women | .06 | -.05 | -.04 | .05 | -.06 | .18 | -.13 |
| *“suicide help”* | Total | -.16 | .09 | .03 | .01 | .12 | .05 | .20 |
|  | Young (<40 yrs) | -.01 | .24 | .21 | .17 | .17 | .11 | **.29*** |
|  | Old (40+ yrs) | -.22 | .07 | -.13 | -.12 | .03 | -.01 | .05 |
|  | Older men | -.08 | -.01 | -.02 | -.10 | .16 | .00 | .07 |
|  | Older women | **-.32**** | -.13 | -.25 | -.05 | **-.27*** | -.02 | -.05 |
| *suicide hotline* | Total | .14 | -.15 | -.14 | -.21 | -.09 | .05 | .16 |
|  | Young (<40 yrs) | .06 | .03 | -.12 | -.13 | -.08 | .14 | **.25*** |
|  | Old (40+ yrs) | .17 | **-.27*** | -.12 | -.20 | -.07 | -.05 | .02 |
|  | Older men | **.30**** | **-.29*** | -.07 | -.14 | -.08 | .16 | -.05 |
|  | Older women | **-.30**** | .08 | -.09 | -.11 | .04 | **-.40**** | .13 |
| *“suicide hotline”* | Total | .07 | .01 | -.05 | .04 | .04 | **.26*** | .05 |
|  | Young (<40 yrs) | .01 | .04 | -.07 | -.02 | -.03 | **.31**** | .04 |
|  | Old (40+ yrs) | .11 | -.01 | -.04 | .07 | .07 | .21 | .05 |
|  | Older men | .15 | -.02 | -.05 | .07 | .04 | .23 | .04 |
|  | Older women | -.08 | .04 | .00 | .07 | .13 | .07 | .06 |
| *suicide prevention* | Total | .16 | .13 | .03 | -.16 | .10 | -.03 | .04 |
|  | Young (<40 yrs) | .01 | .16 | .10 | -.08 | .08 | .14 | .17 |
|  | Old (40+ yrs) | **.22*** | .04 | -.06 | -.16 | .08 | -.17 | -.12 |
|  | Older men | **.31**** | .00 | -.05 | -.09 | .09 | -.04 | -.15 |
|  | Older women | -.19 | .08 | .00 | -.14 | -.04 | **-.28**** | -.07 |
| *“suicide prevention”* | Total | .05 | **.27*** | .01 | -.14 | .03 | -.12 | .02 |
|  | Young (<40 yrs) | -.02 | **.32**** | .06 | -.05 | .03 | -.07 | .12 |
|  | Old (40+ yrs) | .09 | .11 | -.05 | -.16 | .03 | -.13 | -.09 |
|  | Older men | **.24*** | ,07 | -.06 | -.13 | -.02 | .03 | -.12 |
|  | Older women | **-.34**** | .08 | .04 | -.03 | .09 | **-.32**** | .07 |
| *“suicide survivors”* | Total | .22 | .09 | .08 | -.06 | -.03 | .25 | .08 |
|  | Young (<40 yrs) | .19 | -.02 | .07 | .02 | -.02 | .19 | -.04 |
|  | Old (40+ yrs) | .17 | .14 | .06 | -.11 | -.03 | .21 | .15 |
|  | Older men | .21 | .18 | .02 | -.18 | .07 | .23 | .18 |
|  | Older women | -.10 | -.10 | .09 | .15 | -.21 | -.05 | -.07 |

*Note.* * *p* < .05 (two-tailed); ** *p* < .01 (two-tailed). Significant (*p* < .05) cross-correlations are printed boldface.
