# Supplementary material for: Low validity of Google Trends for behavioral forecasting of national suicide rates
Source: PLoS One. 2017 Aug 16;12(8):e0183149. doi: 10.1371/journal.pone.0183149 (PMC5558943; doi:10.1371/journal.pone.0183149)
Supplement: S2 Table — (DOCX) [file pone.0183149.s006.docx]

**S2 Table. Cross-correlations of selected search terms and suicide rates at lags (in months) -3 to +3 in the German data.**

|  |  | Lag (in months) | | | | | | |
| --- | --- | --- | --- | --- | --- | --- | --- | --- |
| Search term | Suicide rates | -3 | -2 | -1 | 0 | +1 | +2 | +3 |
| *Suizid* | Total | .06 | .03 | .09 | .03 | **-.20*** | -.12 | -.07 |
|  | Young (<40 yrs) | .03 | .07 | -.11 | -.01 | -.03 | -.09 | -.04 |
|  | Old (40+ yrs) | .05 | .02 | -.06 | .04 | **-.22*** | -.11 | -.07 |
|  | Older men | .07 | .04 | -.10 | .03 | **-.25**** | -.07 | -.05 |
|  | Older women | -.01 | -.04 | .04 | .04 | -.08 | -.17 | -.09 |
| *Selbstmord* | Total | -.05 | -.02 | .11 | .16 | -.06 | .01 | -.03 |
|  | Young (<40 yrs) | -.15 | .19 | -.02 | .02 | .14 | -.07 | .02 |
|  | Old (40+ yrs) | .01 | -.11 | .14 | .18 | -.13 | .04 | -.04 |
|  | Older men | .01 | -.05 | .13 | .18 | -.12 | .16 | -.03 |
|  | Older women | .01 | -.16 | .10 | .11 | -.10 | -.15 | -.04 |
| *Depressionen* | Total | .01 | -.13 | .07 | .12 | -.04 | .00 | .11 |

|  | Young (<40 yrs) | -.07 | .09 | .10 | -.01 | .15 | -.18 | -.05 |
| --- | --- | --- | --- | --- | --- | --- | --- | --- |
|  | Old (40+ yrs) | .05 | -.19 | -.03 | .15 | -.12 | .09 | .15 |
|  | Older men | .05 | -.11 | .02 | .10 | -.10 | .11 | .18 |
|  | Older women | .03 | **-.25**** | .04 | .16 | -.09 | .02 | .04 |
| *Freitod* | Total | -.15 | -.11 | -.12 | .10 | .08 | .09 | .04 |
|  | Young (<40 yrs) | .01 | -.02 | -.05 | .09 | .06 | .09 | .11 |
|  | Old (40+ yrs) | -.19 | -.13 | -.12 | .09 | .08 | .07 | .01 |
|  | Older men | -.15 | -.11 | -.13 | .07 | .07 | .12 | .05 |
|  | Older women | -.21 | -.13 | -.07 | .10 | .07 | -.08 | -.10 |
| *Selbstmord Forum* | Total | .00 | .08 | .06 | -.02 | .02 | -.02 | -.03 |
|  | Young (<40 yrs) | .01 | -.07 | .04 | .04 | .09 | -.04 | -.03 |
|  | Old (40+ yrs) | .00 | .11 | .06 | -.04 | -.01 | -.01 | -.03 |
|  | Older men | .02 | .11 | .00 | -.04 | -.02 | .00 | -.03 |
|  | Older women | -.04 | .08 | .18 | -.02 | .02 | -.03 | -.01 |

*Note.* * *p* < .05 (two-tailed); ** *p* < .01 (two-tailed). Significant (*p* < .05) cross-correlations are printed boldface.
