# Supplementary material for: Low validity of Google Trends for behavioral forecasting of national suicide rates
Source: PLoS One. 2017 Aug 16;12(8):e0183149. doi: 10.1371/journal.pone.0183149 (PMC5558943; doi:10.1371/journal.pone.0183149)
Supplement: S3 Table — (DOCX) [file pone.0183149.s007.docx]

**S3 Table. Cross-correlations of selected search terms and suicide rates at lags (in months) -3 to +3 in the Austrian data.**

|  |  | Lag (in months) | | | | | | |
| --- | --- | --- | --- | --- | --- | --- | --- | --- |
| Search term | Suicide rates | -3 | -2 | -1 | 0 | +1 | +2 | +3 |
| *Suizid* | Total | .22 | -.06 | -.23 | .12 | .07 | -.25 | .31 |
|  | Young (<40 yrs) | .17 | .07 | -.21 | .20 | -.25 | -.08 | .13 |
|  | Old (40+ yrs) | .17 | -.12 | -.15 | .03 | .24 | -.26 | .31 |
|  | Older men | .20 | -.12 | -.14 | .06 | .17 | -.16 | .32 |
|  | Older women | .03 | -.08 | -.12 | -.04 | .28 | **-.34*** | .17 |
| *Selbstmord* | Total | -.09 | **.32**** | -.02 | .13 | -.05 | -.14 | .00 |
|  | Young (<40 yrs) | -.08 | -.01 | -.02 | .13 | .09 | -.19 | .07 |
|  | Old (40+ yrs) | -.06 | **.38**** | -.01 | .08 | -.12 | -.05 | -.04 |
|  | Older men | -.06 | **.37**** | -.02 | .12 | -.04 | -.04 | -.10 |
|  | Older women | -.02 | **.22*** | .00 | -.03 | -.19 | -.05 | .09 |
| *Depressionen* | Total | -.22 | -.14 | .02 | -.10 | **.31**** | -.04 | -.12 |

|  | Young (<40 yrs) | **-.26*** | -.14 | .08 | -.15 | .13 | **-.23*** | .22 |
| --- | --- | --- | --- | --- | --- | --- | --- | --- |
|  | Old (40+ yrs) | -.10 | -.08 | -.02 | -.04 | **.28*** | .08 | **-.25*** |
|  | Older men | -.10 | -.10 | -.02 | -.06 | **.27*** | .12 | **-.23*** |
|  | Older women | -.07 | .00 | -.01 | .03 | .17 | -.02 | -.19 |

*Note.* * *p* < .05 (two-tailed); ** *p* < .01 (two-tailed). Significant (*p* < .05) cross-correlations are printed boldface.
