# Supplementary material for: Low validity of Google Trends for behavioral forecasting of national suicide rates
Source: PLoS One. 2017 Aug 16;12(8):e0183149. doi: 10.1371/journal.pone.0183149 (PMC5558943; doi:10.1371/journal.pone.0183149)
Supplement: S4 Table — (DOCX) [file pone.0183149.s008.docx]

**S4 Table. Cross-correlations of selected search terms and suicide rates at lags (in months) -3 to +3 in the Swiss data.**

|  |  | Lag (in months) | | | | | | |
| --- | --- | --- | --- | --- | --- | --- | --- | --- |
| Search term | Suicide rates | -3 | -2 | -1 | 0 | +1 | +2 | +3 |
| *Selbstmord* | Total | -.23 | -.15 | -.11 | .19 | -.05 | -.17 | .02 |
|  | Young (<40 yrs) | -.04 | .11 | -.18 | .26 | .07 | -.06 | -.27 |
|  | Old (40+ yrs) | -.23 | -.21 | -.03 | .07 | -.09 | -.15 | .16 |
|  | Older men | -.27 | -.18 | .10 | -.01 | -.16 | -.15 | .15 |
|  | Older women | -.01 | -.13 | -.24 | .19 | .11 | -.07 | .06 |
| *Depressionen* | Total | -.22 | -.19 | .20 | -.05 | .11 | -.07 | .03 |
|  | Young (<40 yrs) | -.23 | -.19 | **.54**** | -.06 | .03 | -.21 | -.04 |
|  | Old (40+ yrs) | -.12 | -.11 | -.04 | -.02 | .10 | .02 | .04 |
|  | Older men | .06 | .00 | -.11 | -.09 | .17 | -.08 | -.11 |
|  | Older women | **-.33*** | -.21 | .10 | .10 | -.09 | .17 | .25 |

*Note.* * *p* < .05 (two-tailed); ** *p* < .01 (two-tailed). Significant (*p* < .05) cross-correlations are printed boldface
